# Sexually deceptive orchids with distinct flower morphologies elicit different behaviours from a shared pollinator

**DOI:** 10.1093/aob/mcaf234

**Published:** 2025-09-26

**Authors:** Marinus L de Jager, Noushka Reiter, Mike Wicks, Björn Bohman, Gareth D Holmes, Ryan D Phillips

**Affiliations:** Department of Ecology, Plant and Animal Sciences, La Trobe University, Melbourne, Victoria 3086, Australia; Science Division, Royal Botanic Gardens Victoria, Cranbourne, Victoria 3977, Australia; Department of Ecology, Plant and Animal Sciences, La Trobe University, Melbourne, Victoria 3086, Australia; Science Division, Royal Botanic Gardens Victoria, Cranbourne, Victoria 3977, Australia; Ecology and Evolution, Research School of Biology, ANU College of Science, Canberra ACT 2600, Australia; Science Division, Royal Botanic Gardens Victoria, Cranbourne, Victoria 3977, Australia; School of Molecular Sciences, The University of Western Australia, Crawley, WA 6009, Australia; Department of Plant Protection Biology, The Swedish University of Agricultural Sciences, Lomma 23422, Sweden; Department of Ecology, Plant and Animal Sciences, La Trobe University, Melbourne, Victoria 3086, Australia; Science Division, Royal Botanic Gardens Victoria, Cranbourne, Victoria 3977, Australia; Department of Ecology, Plant and Animal Sciences, La Trobe University, Melbourne, Victoria 3086, Australia; Science Division, Royal Botanic Gardens Victoria, Cranbourne, Victoria 3977, Australia

**Keywords:** Endangered species, fitness, hybridisation, interspecific pollen transfer, mating behaviour, nectar, pollination efficiency, seed viability, specialization, sucrose

## Abstract

**Background and Aims:**

Pollination by sexual deception is one of the most specialized pollination strategies among angiosperms, with co-occurring plant species often exploiting males of different insect species. We test if the morphologically divergent orchids *Caladenia cardiochila* and its sympatric endangered congener *C. lowanensis* are dependent on the same thynnine wasp pollinator. We further investigate the role of floral traits on pollinator behaviour and evaluate potential hybridization risk.

**Methods:**

Pollinator sharing was tested for with DNA barcoding. Pollinator behaviour was quantified and experimental floral dissections were used to determine the site of sexual attractant release. We employed GC–MS to test for the presence of sugar on orchid labella, hand crosses to assess the impact of interspecific pollen transfer on seed viability, and population monitoring to quantify natural pollination success.

**Key Results:**

We found that *C. cardiochila* and *C. lowanensis* both employ sexual deception of *Phymatothynnus* aff. *nitidus* wasps as a pollination strategy. However, the behaviour they elicit differs, with wasps attempting to mate with the insectiform labellum in *C. cardiochila* and the glandular sepal tips in *C. lowanensis*, which are the respective sources of sexual attractant. Unlike most sexually deceptive orchids, *C. lowanensis* secretes minute amounts of sugar from its labellum. While wasps interacted more frequently with the labellum in *C. cardiochila*, placing them closer to its reproductive structures, both species exhibited comparable pollination success and pollen transfer efficiency. Experimental crosses revealed that hybrid seed has high viability.

**Conclusions:**

Sexual deception of the same pollinator by orchids varying in the location of sexual attractant and flower morphology highlights the considerable flexibility of this pollination strategy. Given their overlapping distributions and the viability of hybrid seed, pollinator sharing poses a hybridization risk that needs to be considered in the management of wild *C. lowanensis* populations and future conservation translocations.

## INTRODUCTION

Reproduction in flowering plants is primarily dependent on animals moving pollen between conspecific flowers (Ollerton *et al.*, 2011). This reliance on pollinators has produced a multitude of floral adaptations that promote animal-mediated pollen transfer (Harder and Johnson, 2009; Minnaar *et al.*, 2019). Many flowering plants have specialized on a small subset of the potential pollinators available to them (Fenster *et al.*, 2004). Several morphological (e.g. nectar tube length; Pauw *et al.*, 2009; flower colour; Newman *et al.*, 2012) and chemical (e.g. volatile floral emissions; Bohman *et al.*, 2016) adaptations contribute to pollinator specialization and floral trait diversity. When plants share the same pollinators, specialization often leads to floral trait convergence (Fenster *et al.*, 2004; Rosas-Guerrero *et al.*, 2014), although variation in traits also occurs (Phillips *et al.*, 2013; Ellis *et al.*, 2014; Kopper *et al.*, 2024).

Within the flowering plants, the Orchidaceae are an exemplar of floral adaptation and pollinator specialization (Tremblay *et al.*, 2005; Johnson and Schiestl, 2016), with many species employing a single pollinator (Ackerman *et al.*, 2023). In orchids, specialization arises via visual (de Jager *et al.*, 2016; Sletvold *et al.*, 2016) and/or olfactory (Schiestl *et al.*, 2003; Hetherington-Rauth and Ramírez, 2016) cues that target specific pollinating species, as well as through precise morphological fit of the pollinator between the labellum and column (Johnson and Steiner, 1997; de Jager and Peakall, 2019). One of the most specialized pollination strategies among orchids is sexual deception (Ackerman *et al.*, 2023; Peakall, 2023), which involves the sexual attraction of male insects (Peakall, 1990) through precise chemical (Schiestl *et al.*, 2003) and often morphological (de Jager and Peakall, 2016) mimicry of conspecific females. Although all sexually deceptive plants rely on attracting mate-seeking male insects, there is considerable variation in how these flowers operate. In some species pollen removal is dependent on male pollinators attempting to copulate with the labellum (de Jager and Peakall, 2019), while in others pollen transfer occurs during pre-mating courtship behaviour (Peakall, 1990) or when male pollinators become temporarily trapped inside flowers (Phillips *et al.*, 2014).

While pollination by sexual deception occurs in Europe, South and Central America, Asia and Africa, one of the greatest centres of diversity is Australia, where hundreds of orchid species across 11 genera use this pollination strategy (Peakall, 2023). Of these orchid genera, *Caladenia* is the largest, with over 395 species and subspecies described (Backhouse, 2018). Although some *Caladenia* species are pollinated by nectar-seeking bees and wasps (Reiter *et al.*, 2018, 2019*a*, *b*; Phillips *et al.*, 2021), over 200 species in the genus are predicted to be pollinated by sexual deception of thynnine wasps (Phillips *et al.*, 2017). An exceptional characteristic of *Caladenia* is the striking floral variation found among these sexually deceptive species, which range from species bearing small insectiform flowers to species with large colourful flowers lacking any insectiform features. Although the function of floral traits in insectiform *Caladenia* has not been experimentally studied, observations of pollinator behavioural interactions (Stoutamire, 1974, 1983) suggest their elaborate labella may represent morphological mimics of a female wasp, as found in eastern Australian sexually deceptive *Chiloglottis* orchids (de Jager and Peakall, 2016).

In contrast, many large-flowered sexually deceptive *Caladenia* exhibit thickened glandular sepal or petal tips with swollen osmophores that are the source of volatile sexual attractants (Phillips *et al.*, 2013, 2024; Phillips and Peakall, 2018; Reiter *et al.*, 2023). Some species of *Caladenia* have recently been demonstrated to exploit sexual behaviour as well as feeding behaviour of their pollinators (Phillips *et al.*, 2024*a*). This strategy appears to be rare in orchids but is well documented in several populations of the sexually deceptive daisy *Gorteria diffusa* (Ellis and Johnson, 2010; de Jager and Ellis, 2013, 2014). Based on the discovery of this strategy in multiple species complexes in *Caladenia* (Phillips *et al.*, 2024*a*), it may prove to be phylogenetically widespread in the genus. The variation that *Caladenia* exhibits in strategies for pollinator attraction, as well as floral morphology, make it an effective genus for exploring how floral traits impact pollinator behaviour and pollination success.

While related species of sexually deceptive orchids often exploit different pollinator species (Paulus, 2006; Phillips *et al.*, 2009; Peakall *et al.*, 2010), pollinator sharing between sexually deceptive *Caladenia* species has been reported (Phillips *et al.*, 2017). A shared thynnine wasp pollinator was also documented between different orchid genera, involving the sexually deceptive *Caladenia pectinata* and *Drakaea livida* in Western Australia (Phillips *et al.*, 2013). This comparison revealed that floral traits such as site of odour release can affect pollinator behaviour, which in turn affects pollination success. Such cases where different orchid species use the same pollinator offer a control for inherent biases or preferences within a given pollinator, thereby yielding powerful tests of pollinator responses towards different floral traits. While the use of different sexually deceived pollinators can generate strong reproductive isolation (Xu *et al.*, 2011; Whitehead and Peakall, 2014), pollinator sharing may lead to hybridization and species displacement (Stökl *et al.*, 2008) and is therefore potentially of conservation concern when occurring in rare species (Levin *et al.*, 1996). While pollinator sharing and hybridization may not pose a threat to many orchids due to pre-pollination or genetic barriers (Cozzolino *et al.*, 2006; Gaskett, 2012), it has been implicated in the decline of several endangered orchids (Stökl *et al.*, 2008; Paul *et al.*, 2013). Interspecific pollen transfer may be frequent in some *Caladenia* as hybrids are often represented in herbarium material (Hopper, 2018) and some hybrid combinations are regularly encountered in the field (Backhouse, 2018). Coupled with prior observations of pollinator sharing among threatened *Caladenia* species (Phillips *et al.*, 2009, 2017; Swarts *et al.*, 2014), the potential for hybridization needs to be accounted for in the conservation management of endangered *Caladenia*. This is critical given that no other orchid genus in Australia has more threatened species than *Caladenia* (Dixon and Hopper, 2009), with 73 species currently listed as nationally threatened (*Australian* Government, 2025).

Here, we focus on the endangered *C. lowanensis* and the common congener *C. cardiochila*, which belong to *Caladenia* subgenus *Calonema*. These species occasionally co-occur and putative hybrids with intermediate floral traits have been reported (Backhouse, 2018; VicFlora, 2025). As both species attract sexually deceived thynnine wasps of the genus *Phymatothynnus* (Bates, 1996; Swarts *et al.*, 2014), it is possible they may share the same pollinator. While the potential for pollinator sharing and hybridization between these species is of concern for the conservation of *C. lowanensis*, it also provides an ideal opportunity to examine the influence of floral variation on pollinator behaviour. Here, we investigate the pollination biology and pollination success of both species. Specifically, we ask (1) are they dependent on pollinators for reproduction? (2) do they share pollinator species, and if so (3) can interspecific pollen transfer produce viable hybrid seed? We further investigate (4) their pollination strategies and whether they elicit similar pollinator behaviours, and (5) whether they exhibit comparable natural pollination success.

## MATERIALS AND METHODS

### Study species

**Fig. 1.**
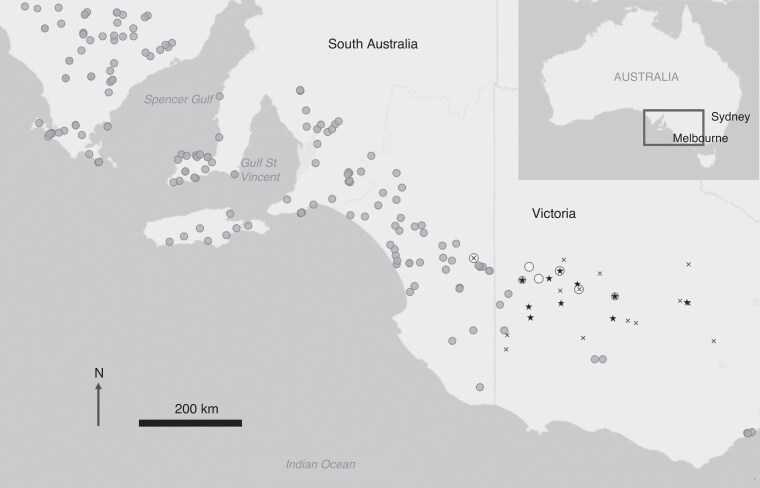
The distribution of *C. cardiochila* (grey circles) and *C. lowanensis* (white circles) in south-eastern Australia. Stars indicate sites where pollinators responded to flowers of *C. cardiochila* and *C. lowanensis* and X represents sites where pollinators were not detected when baiting with flowers.

*Caladenia* species are perennial geophytes that are typically dormant over summer and autumn, emerging in late autumn to early winter as a solitary basal leaf (Backhouse, 2018). Plants produce a single flowering scape per year, with one or a few flowers. The flowers bear sessile or stalked callus structures that are arranged in rows or crowded together on the labellum (VicFlora, 2025). *Caladenia cardiochila* occurs across much of South Australia and western Victoria (Fig. 1), growing in a variety of habitats including coastal heaths, eucalypt woodlands and mallee scrubs on sandy to clay soils (Backhouse, 2018; VicFlora, 2025). Flowering typically occurs from August to October (Jones, 2021). *Caladenia cardiochila* typically bears one, sometimes two, small red and cream flowers. Perianth segments are up to 20 mm long and the insectiform and smooth-lipped cordate labellum bears a dense section of two to four rows of large club-shaped black calli that terminate before the labellum tip (Tate, 1887). Its short sepals and petals are streaked with red lines and lack swollen osmophores on the sepal trips, which occur in many other *Caladenia* species that employ sexual deception. It is known to hybridize with other *Caladenia* species from subgenus *Calonema* (Backhouse, 2018).

*Caladenia lowanensis* is listed as endangered under the Australian *Environment Protection and Biodiversity Conservation Act 1999* and considered Critically endangered employing IUCN criteria under the Victorian State *Flora and Fauna Guarantee Act 1988*. *Caladenia lowanensis* is known from only four populations in Victoria and two in South Australia (Todd, 2000) near the eastern margin of the geographic range of *C. cardiochila* (Fig. 1). It occurs in open dry woodland dominated by *Eucalyptus leucoxylon* on sandy-loam soils (Carr, 1991). It bears a much larger flower than *C. cardiochila* with spreading perianth segments up to 40 mm long. It produces one, or occasionally two, flowers from September to October that are cream to greenish yellow with red striations and red fringed labella containing four to six sparse rows of thin and slightly hooked purple calli. The elongated sepals of *C. lowanensis* taper before terminating in thickened, dark purple, swollen osmophores (hereafter termed ‘clubs’). The pollinator of *C. lowanensis* has previously been reported as the thynnine wasp *Phymatothynnus nitidus* (Swarts *et al.*, 2014), although these wasps were not DNA-sequenced. Reports of occasional hybrids suggest it may share a pollinator with *C. cardiochila* (Backhouse, 2018). However, DNA barcoding has revealed cryptic variation within *P. nitidus* (Reiter *et al.*, 2023), indicating it is likely a complex of species where genetic analysis is required to confirm pollinator sharing. Thynnine wasps are characterized by flightless females that release sex pheromones to attract the flying males (Schiestl *et al.*, 2003; Bohman *et al.*, 2014), who carry females to a food source *in copula* (Alcock, 1981; Brown and Phillips, 2014). Food sources include nectar and sugar exudates from scale insects, leafhoppers and aphids (Brown and Phillips, 2014), although the reliance on nectar varies between species or genera (Menz *et al.*, 2015).

### General pollination methods

From 2022 to 2024 we investigated the pollination biology of *C. cardiochila* and *C. lowanensis.* Pollinator observations were undertaken between 0900 and 1600 h on 30 days with temperatures over 18 °C, which is when thynnine wasps are most active (Peakall, 1990). For all pollinator observations and experiments we used the pollinator baiting method (Stoutamire, 1983; Peakall, 1990), where flowering scapes are moved to a new position in the landscape, leading to a rapid response by sexually deceived male thynnine wasps. Experimental pollinator observations and flower dissections were conducted at the MGC natural bushland site. In addition, pollinator surveys were also carried out with *C. cardiochila* and *C. lowanensis* flowers at wild populations of both species, as well as at suitable habitat where the orchids were not present across 23 locations in Victoria (Fig. 1, Supplementary Data Table S1) to determine how widespread the pollinator is within the study region. Where pollinators were observed visiting bait flowers, their behaviour at flowers was recorded and they were physically captured if possible. For *C. cardiochila* we sourced flowers from wild plants at the MGC (voucher specimen MEL2554486) and the BDB site (MEL2554487). For the endangered *C. lowanensis* we used cut flowers from the Royal Botanical Gardens, Victoria (MEL2554488, MEL2554489), where a population of *C. lowanensis* plants is maintained in a shade house as an *ex situ* conservation collection. These plants were previously propagated from seed obtained by hand-pollinating wild *C. lowanensis* plants at the KFF and WWFF sites. Plants were grown symbiotically from seed with the fungus *Serendipita australiana* (family Serendipitaceae within order Sebacinales) using established methods (Reiter *et al.*, 2016). Flowers were kept fresh at 4 °C in a portable refrigerator between experiments.

### Are *C. cardiochila* and *C. lowanensis* dependent on insects for pollination?

To determine whether *C. cardiochila* can set seed without pollinators, we experimentally excluded insects from 15 plants bearing flower buds at MGC during the 2022 flowering season. Three wooden stakes were driven into the soil 10 cm away from each plant to avoid any damage, and fine white netting with 2-mm mesh was wrapped around and stapled to the wooden stakes without touching the flower buds but covering the entire plant so flying insects could not gain entrance. The netting was removed 4 weeks later, and flowers were observed for either pollen deposition or signs of clear ovary swelling relative to younger flowers, which would indicate fertilization. For *C. lowanensis*, we tagged buds on 15 plants at the *ex situ* collection where insects could not gain access and monitored flowers after 4 weeks for signs of fruit formation.

### Do *C. cardiochila* and *C. lowanensis* share pollinators?

To determine if *C. cardiochila* and *C. lowanensis* share pollinators, we conducted extensive fieldwork as well as pollinator surveys with both species in 2022, 2023 and 2024. We placed flowering scapes bearing a single flower each in water-filled vials (50 mL) attached to bamboo skewers that were stuck into the ground and recorded all visits to flowers. For fieldwork at the MGC site, two scapes of each species were observed by a single observer for 3 min before moving the scapes to a new baiting position at least 5 m away (*n* = 326 trials; Supplementary Data Table S1). This was done as the number of interactions of thynnine pollinators with sexually deceptive orchids are high initially but rapidly decline within the first 4 min at a given location (Whitehead and Peakall, 2013). Repeatedly moving bait flowers across the site ensured continued responses and allowed us to comprehensively cover the local thynnine pollinator population, which can vary over small spatial scales of ≤5 m (Peakall and Beattie, 1996; Menz *et al.*, 2013). A baiting position was not sampled again on the same day, as *Neozeleboria* thynnine wasps have been shown to quickly learn to avoid a given location containing sexually deceptive orchids, but this avoidance appears not to be retained for more than 24 h (Whitehead and Peakall, 2013). For the pollinator survey, a single scape bearing one flower was similarly observed by a single observer for 10 min at a given location before moving ≥200 m to sample pollinators over larger areas of ≥1 km (*n* = 225 trials; Supplementary Data Table S1). For pollinator observations at the MGC site and pollinator survey sites we recorded if and where insects landed on flowers, whether pollinia were removed or deposited, and what behaviour was displayed on flowers (see below). Bait flowers were replaced with fresh flowers as needed.

### Pollinator identification

For the insect species most frequently responding to each *Caladenia* species, we caught 41 individuals and placed them in ethanol for later DNA barcoding (Supplementary Data Table S2). The mitochondrial *COI* region is effective at separating closely related thynnine wasp species that are often similar morphologically (Griffiths *et al.*, 2011), such as in the *Phymatothynnus nitidus* complex (Reiter *et al.*, 2023). Non-destructive DNA isolation was performed using a previously described protocol for gnats (Hayashi *et al.*, 2021) with the per sample volumes of TNES lysis buffer and proteinase K increased to 300 and 35 µL respectively. The resultant DNA pellets were dissolved in 150 µL 10 mm Tris–EDTA buffer and the DNA concentration of representative samples was checked using a Qubit v3 fluorometer with a dsDNA HS kit (Invitrogen). PCR amplification of a portion of *COI* was performed in 20-µL reactions containing 10 µL 2× MyTaq Red Mix (Bioline), 0.5 µL of each 10 µm primer (LCO1490 and HCO2198; Folmer *et al.*, 1994), 1.5–2 µL DNA isolate and sterile distilled water to volume. For samples that amplified poorly on the first attempt an extra 0.6 µL of 25 mm MgCl_2_ was included in the reactions. Amplification was performed on an Eppendorf Nexus Cycler with a protocol consisting of 5 min denaturation at 95 °C followed by 35 cycles of 30 s at 95 °C, 30 s annealing initially at 62 °C reducing 3 °C each cycle to 44 °C, a 45-s elongation at 72 °C, and then a final step of 7 min at 72 °C. DNA amplicons were bidirectionally Sanger-sequenced by AGRF, Melbourne, using the same primers as used for PCR.

Raw chromatogram data were checked and assembled using the program Geneious Prime v2023.2.1 and consensus sequences generated and aligned using the standard Geneious algorithm. To screen for the potential presence of pseudogenic *COI* copies, the consensus sequences were translated to amino acids against all possible codon frames using the invertebrate mitochondrial translation table as implemented in Geneious Prime. Any sequences containing unexpected stop codons after translation using the correct frame were excluded from downstream analyses. A multiple sequence alignment was conducted before estimating the genetic variation based on percentage variation in the number of base pairs within the *COI* region of each sample. Previous work on thynnine wasps has shown that genetic variation within a species is typically 3–5 % (Phillips *et al.*, 2017). Wasps sequenced in this study were also compared with the sequences of wasps pollinating the congeners *C. xanthochila*, *C. fitzgeraldii* and *C. robinsonii*.

### Does interspecific pollen transfer yield viable seed?

To determine if the endangered *C. lowanensis* can produce viable seeds from interspecific crosses with *C. cardiochila*, we collected pollinia from *C. cardiochila* flowers at the MGC site (*n* = 28 flowers, 28 plants) in 2024. This was done by swiping a clean toothpick under the anther cap and transferring the collected pollinia to an airtight container for later use. The plants of *C. lowanensis* used in our pollinator experiments were from the *ex situ* collection. A pollinium of *C. cardiochila* was smeared across a receptive stigma of *C. lowanensis* until deposited pollen was visible on the stigma (*n* = 28 flowers, 28 plants). Using the same technique on the same day, we also collected pollinia from *C. lowanensis* in the *ex situ* shade house and applied it to the receptive stigmas of *C. lowanensis* flowers on different plants (*n* = 29 flowers, 29 plants). Flowers that were pollinated were individually bagged with small mesh bags. Capsules were harvested when dry, emptied into seed packets, and kept at 15 % relative humidity for 10 d before sealing packets for storage at −20 °C.

**Fig. 2.**
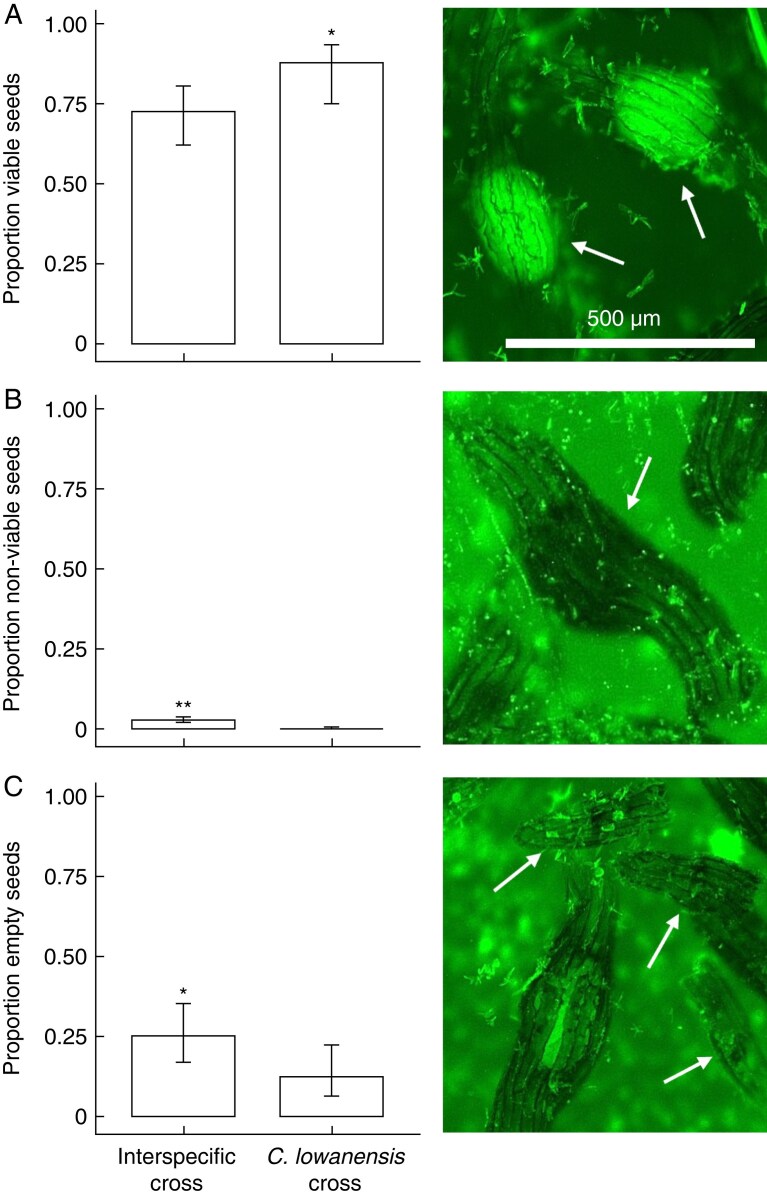
The proportion of (A) seeds with viable embryos, (B) seeds with embryos that were not viable and (C) empty seeds lacking an embryo from interspecific crosses between *C. cardiochila* and *C. lowanensis* and pure *C. lowanensis* crosses. Arrows in the photographs indicate each seed type. *P < 0.05; **P < 0.005.

To quantify seed viability, we used a modified version of the method of Pritchard (1985). We first added seeds to 500 µL deionized water containing 0.002 % Tween 20 for 24 h. After this rehydration period, seeds were added to slides and stained for 15 min with a 60-µL solution comprising 0.5 % (w/v) fluorescein diacetate (FDA) dissolved in absolute acetone (Widholm, 1972). Slides were viewed with an FITC filter on an Olympus BX53 Digital Upright Microscope using a 100-W LED and U-LGPS fluorescence light source that excites fluorescein at 480 nm and collects emission at 535 nm. Seeds were then visualized with cellSans Standard 4.3.1 software using a DP23M camera. Three counts of ∼100 seeds were counted to yield ∼300 seeds scored per cross for seed viability. Viable seeds display bright greenish yellow embryos using this method (Pritchard, 1985; Fig. 2), while seeds with non-viable embryos do not fluoresce and empty seeds lacking an embryo are detected by the presence of a seed coat.

### Do *C. cardiochila* and *C. lowanensis* elicit different responses from their pollinators?

For each insect responding to a flower at the MGC site and pollinator survey sites we recorded whether it landed, on which floral part it alighted, and whether it made contact with the column, the structure that contains both anthers and stigma. For visitors that touched the column, we recorded whether they deposited or removed pollinia and the behaviour associated with deposition or removal. Attempted mating behaviour by thynnine wasps towards sexually deceptive orchids is evident by arching of the abdomen to curl the abdomen tip repeatedly towards the floral surface, referred to as pseudocopulation (Stoutamire, 1974). This behaviour is analogous to mating behaviour of male thynnids when they locate a receptive wingless female before they fly off with her *in copula* (Alcock, 1981). We noted where attempted pseudocopulation was displayed on *C. cardiochila* and *C. lowanensis* flowers. In addition, we also recorded any non-mating behaviour on the labellum that did not include mating behavioural routines, but that could potentially contribute to pollen transfer.

### What is the site of odour release?

**Fig. 3.**
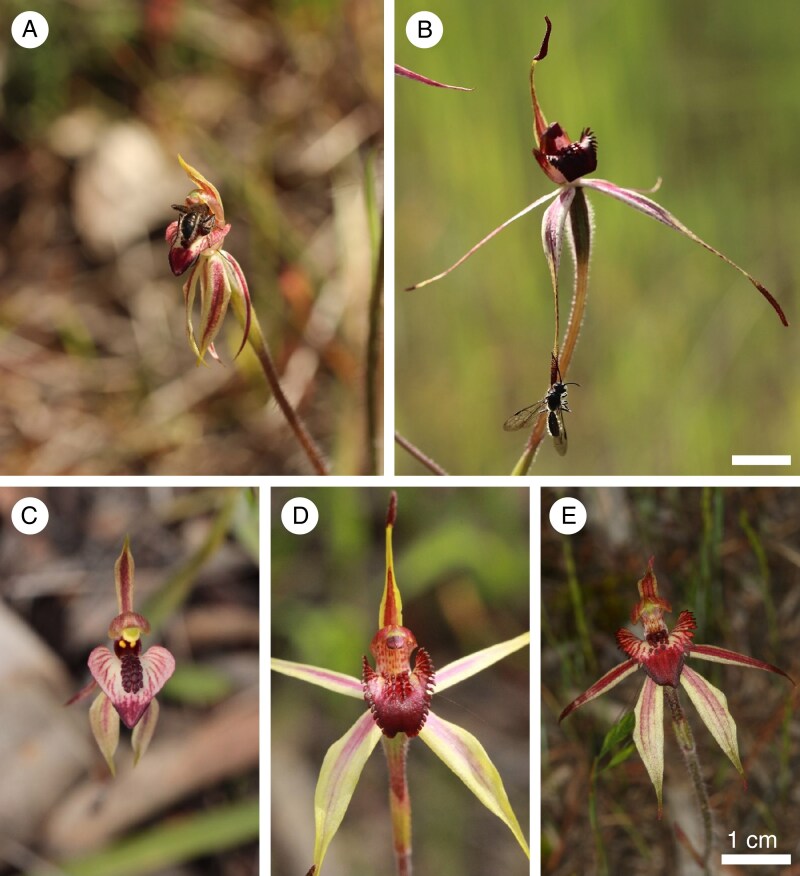
Male *Phymatothynnus* aff. *nitidus* wasp pollinators displaying mating behaviour on the (A) *C. cardiochila* labellum and (B) *C. lowanensis* sepal clubs. See Supplementary Data Videos S1 and S2 for further details of these behaviours. Flower close-ups of (C) *C. cardiochila*, (D) *C. lowanensis* and (E) a putative hybrid between these species. Note the mix of floral traits in the hybrid flower, including a semi-fringed labellum, intermediate insectiform calli and diminished sepal clubs. Scale bar = 1 cm. Photographs: Marinus de Jager (A–D), Len and Marie-Josée Carrigan (E).

To investigate which parts of the flower are the source of sexual attractant in *C. cardiochila* and *C. lowanensis*, we dissected individual flowers into the column, labellum, sepal tips, petal tips and the remains of the flower, termed the floral display. Each floral part was attached to a bamboo skewer with a dressmaker’s pin. In a preliminary test, we presented each of the floral parts of a given species simultaneously, with each bamboo skewer separated by 15 cm. This revealed that the labellum is likely the most attractive floral part in *C. cardiochila*, and the sepal tips the most attractive in *C. lowanensis*, as the first ten visitors to each species preferentially landed on those floral parts, respectively. Subsequently, we conducted a replicated dissection experiment in two presentation phases. In the initial phase we presented the least attractive floral parts together for 2 min, while the more attractive parts were concealed in an airtight container. For the second phase we added the most attractive floral part for each species to the remainder of the floral parts for another 2 min. The initial phase aims to determine if any additional floral parts are attractive to pollinators, besides the dominant attractive floral part, while the second phase acts as a positive control to confirm that pollinators are present and responsive. Due to differences in the availability of fresh flowers at the time of respective dissection experiments, we conducted eight replicates for each of five individual flowers for *C. cardiochila* and five replicates for each of eight individual flowers for *C. lowanensis*. In a follow-up experiment with *C. cardiochila*, we dissected the tip from the remainder of the labellum, as it has a thickened red margin of additional callus tissue (Fig. 3A) that may be important for sexual attraction (Wong *et al.*, 2017; de Jager and Peakall, 2019). For six flowers, the labellum tip and labellum remainder were pinned to separate bamboo skewers, which were presented simultaneously 50 cm apart for 2 min.

### Do *C. cardiochila* and *C. lowanensis* provide any sugar?

To determine whether *C. cardiochila* and *C. lowanensis* provide sugar on the surface of the labellum, we used a method designed to detect minute quantities of sugar in closely related *Caladenia* (Reiter *et al.*, 2018, 2019*a*, *b*; Phillips *et al.*, 2024*a*). We selected ten fresh flowers on separate plants on warm days in the field. For each flower, we added 5 µL aqueous solution of ribitol (internal standard, 0.2 mg mL^−1^) to the labellum with a glass syringe. This solution was added to the calli and therefore covered a section of the labellum, representing the area where most visitors direct their attention when landing on the labellum. We collected this aqueous extract from the labellum with 5-µL microcapillary tubes and immediately transferred it to 2-mL GC vials with 50-µL inserts. This was repeated three times for each flower on different parts of the labellum calli, and the combined extracts from each flower were transferred to a single GC vial. Extracts were stored on ice in the field and then kept in a −20 °C freezer until analysis following the methods of Reiter *et al.* (2018).

### What is the pollination success of *C. cardiochila* and *C. lowanensis* in the field?

From 2022 to 2024 all *C. cardiochila* flowers at the MGC (35 m × 45 m area) and BDB (80 m × 90 m) sites were inspected for pollination and all *C. lowanensis* flowers at the KFF site (30 m × 30 m area), which was the only wild site where flowers were detected. For each flower we recorded evidence of at least one pollinium being removed, as well as evidence of pollen deposition determined by either pollen visible on the stigma or clear signs of ovary swelling following pollination. For each site in each year, we calculated the percentage of flowers with pollinia removed (number of flowers with one or both pollinia missing/total number of flowers inspected), the percentage of flowers pollinated (number of flowers with pollen deposited or ovary swelling/total number of flowers inspected), and the pollen transfer efficiency (percentage of flowers pollinated/percentage of flowers with pollinia removed), which ranges from 0 to 1.

### Data analyses

To test for differences in capsule formation between pure *C. lowanensis* crosses and *C. cardiochila* × *C. lowanensis* interspecific crosses, we used a series of general linear models (GLMs) with a binomial distribution and logit link function. To assess differences in the number of viable seeds, non-viable seeds, and empty seeds produced by pure *C. lowanensis* crosses and *C. cardiochila* × *C. lowanensis* interspecific crosses we used a series of general linear models (GLMs) with a quasibinomial distribution and logit link function as our seed data showed evidence of overdispersion.

To investigate differences between pollinator behaviour on *C. cardiochila* and *C. lowanensis* flowers, we employed generalized linear mixed models (GLMMs) with a binomial distribution and logit link function using the lme4 package version 1.1-35.1 (Bates *et al.*, 2015). To determine if there were significant differences between pollinators landing on each species or displaying a given behaviour on each species, we used the *Caladenia* species a wasp responded to as a fixed factor, with the baiting location in the landscape given a unique number and used as a categorical random factor to account for potential differences in environmental conditions between baiting positions. For our dissection experiment aiming to determine the source of attractant in *C. cardiochila* and *C. lowanensis*, we used a GLMM with a binomial distribution and logit link function to test whether the likelihood of a pollinator landing on a given floral part (i.e. labellum) differed between the two species. We only used data from the second presentation when all floral parts were presented together and used the *Caladenia* species as the fixed factor and the dissected flower number as the random variable to account for any potential differences between flowers. For the experiment where we tested for any difference in attraction between the labellum parts in *C. cardiochila*, we similarly used a GLMM with a binomial distribution and logit link function with the floral part (labellum tip vs rest of labellum) as the fixed factor and the dissected flower number as the random factor. To explore residuals and check for overdispersion of our data we used the package *DHARMa* version 0.4.6 (Hartig, 2022). All statistical analyses were conducted in R 4.3.2 (R Core Team, 2016).

## RESULTS

### Are *C. cardiochila* and *C. lowanensis* dependent on insects for pollination?

Only one of 15 caged and netted *C. cardiochila* flowers at the MGC site displayed evidence of ovary swelling indicative of pollination, likely due to the flower growing against the netting material that can dislodge a pollinium and lead to self-pollination. None of the 15 *C. lowanensis* plants in the *ex situ* shade house collection displayed signs of fertilization, confirming that neither species can autonomously self-pollinate and both require pollen vectors to reproduce successfully.

### Do *C. cardiochila* and *C. lowanensis* share pollinators?

Pollinators responded to flowers at 10 of the 24 sites that were baited with flowers from 2022 to 2024 (Fig. 1). We caught wasps during pollinator observations at the main fieldwork site (MGC), as well as at additional survey sites within orchid habitat, including wild populations of both *C. cardiochila* and *C. lowanensis*. This yielded 14 wasps caught on *C. cardiochila* and 27 wasps caught on *C. lowanensis* across eight sites (Supplementary Data Table S2). For these wasps the mitochondrial *COI* region revealed an average nucleotide divergence of 1.58 % (range 0–4.10 %, *n* = 41), indicating that all samples are from the same species (Phillips *et al.*, 2017), namely *Phymatothynnus* aff. *nitidus*. In contrast, the average nucleotide divergence between the wasps we caught on *C. cardiochila* and *C. lowanensis* and *Phymatothynnus* wasps caught on other *Caladenia* species (Reiter *et al.*, 2023) was much greater (22.2 % divergence to wasps caught on *C. xanthochila* (*n* = 7), 20.8 % to wasps caught on *C. fitzgeraldii* (*n* = 3), and 9.4 % to wasps caught on *C. robinsonii* (*n* = 3)).

### Does interspecific pollen transfer yield viable seed?

Capsule formation was similar for interspecific crosses between *C. cardiochila* and *C. lowanensis* (89.3 %, *n* = 28) and pure *C. lowanensis* crosses (96.6 %, *n* = 29) (GLM, *z* = 1.021, *P* = 0.307). Inspection of FDA-stained seed under a fluorescent microscope revealed that pure *C. lowanensis* crosses (*n* = 19) had significantly more seeds with viable embryos (87.9 %) than interspecific crosses (*n* = 24, 72.5 %) (Fig. 2A; GLM, *t* = 2.389, *P* = 0.022). Seeds with non-viable embryos were more frequent in interspecific crosses (2.6 %) than in pure crosses (0.1 %) (Fig. 2B; GLM, *t* = −3.551, *P* < 0.005), as were empty seeds lacking an embryo (interspecific crosses 24.8 %, pure crosses 12.1 %) (Fig. 2C; GLM, *t* = −2.029, *P* = 0.049).

### Do *C. cardiochila* and *C. lowanensis* elicit different responses from their pollinators?

A total of 593 insects responded to intact flowers across 326 three-minute fieldwork trials and 225 ten-minute pollinator survey trials (Supplementary Data Table S1). Of these insects, 292 responded to *C. cardiochila* and 301 to *C. lowanensis*. The vast majority were male wasps of *Phymatothynnus* aff*. nitidus* (99 % of visits to *C. cardiochila* and 98 % of visits to *C. lowanensis*). The few additional visitors were small Proctotrupidae parasitoid wasps, which were not of suitable size and did not show suitable behaviour to achieve pollination. The *P.* aff. *nitidus* males displayed the zigzag flying approach to flowers characteristic of thynnine wasps following an odour plume in other sexually deceptive orchids (Stoutamire, 1983; Peakall, 1990; Phillips *et al.*, 2009).

Our baiting experiments showed that there was no significant difference between the probability of males landing on (GLMM, *z* = −1.736, *P* = 0.083), or displaying mating behaviour (GLMM, *z* = −1.174, *P* = 0.241) on *C. cardiochila* and *C. lowanensis* flowers. The location where males alighted and where they displayed mating behaviour, however, differed substantially (Table 1). Over 80 % of wasps landing on *C. cardiochila* alighted on the labellum compared with only 41 % in *C. lowanensis*. In contrast, only 14 % of males that landed on *C. cardiochila* alighted on the tepals, while 57 % of landing males did so on *C. lowanensis*. This pattern was mirrored in the location of mating behaviour. All males that displayed mating behaviour on *C. cardiochila* did so on the labellum (Fig. 3A, Supplementary Data Video S1), where they either probed the tip of the labellum or the edge of the callus structures. In contrast, on *C. lowanensis* all but one male displayed mating behaviour on the sepal clubs (Fig. 3B, Supplementary Data Video S2), where males would grasp the sepals and attempt copulation by repeatedly probing the sepal club.

**Table 1.** Pollinator responses and behaviour on *C. cardiochila* and *C. lowanensis* flowers across all sites detailing the location where they landed and displayed mating behaviour, as well as behaviour related to pollen transfer.

Behaviour	*C. cardiochila*	*C. lowanensis*
Total wasp responses	289	294
Landing wasps	226	212
On labellum	182 (80.5 %)	87 (41.0 %)
On sepals or petals (tepals)	31 (13.7 %)	120 (56.6 %)
On column	13 (5.8 %)	5 (2.4 %)
Mating responses	45	25
On labellum	45 (100 %)	1 (4.0 %)
On sepal clubs	0 (0.0 %)	24 (96.0 %)
Non-mating responses on labellum calli	0	14
Wasps that contacted the column	51	14
Wasps arriving with pollinia attached	12	12
Landing on labellum	11	2
Landing on tepals	1	5
Wasps that removed a pollinium	5	1
Wasps that deposited pollen	1	1

On *C. cardiochila* flowers, pollinators sometimes contacted the column (*n* = 51 of 226 wasps that landed), but pollinia removal (*n* = 5 of 226 wasps) was only associated with mating behaviour where wasps grabbed the calli and probed the labellum tip or the calli. Pollinia removal typically only occurred when male wasps made extended contact with the column and often became momentarily stuck. This led to the wasp vigorously trying to free itself and twisting out of position, thereby pulling the pollinium free of the column where it attached to the dorsal side of the wasp’s thorax (Supplementary Data Video S3). Twelve wasps landed on *C. cardiochila* flowers with pollinia already attached to their thorax. While 11 of these landings were on the labellum, only one wasp displayed mating behaviour on the labellum and deposited pollen (Table 1). The pollinia observed on the thorax of pollinators were most likely from *C. cardiochila* flowers, which were the most common *Caladenia* species flowering where pollen transfer was observed (MGC site). Flowering *C. xanthochila* was also present in lower numbers, but it is pollinated by a different species of thynnine wasp (Reiter *et al.*, 2023).

On *C. lowanensis* flowers, most pollinator activity and mating behaviour was directed to the sepal tips. The males that landed on the labellum of *C. lowanensis* showed no evidence of attempting to copulate with the labellum, as they do on *C. cardiochila* flowers. Instead, some males exhibited a prolonged focus on the vicinity of the calli on the labellum lamina, with the head and mouthparts contacting the labellum surface. This non-mating behaviour did not include typical pseudocopulation routines like abdomen curling (Stoutamire, 1974) and was similar to behaviour associated with the search for food resources, as recently documented in other sugar-providing sexually deceptive *Caladenia* (Phillips *et al.*, 2024*a*). Pollinators contacted the reproductive column in *C. lowanensis* (*n =* 14) less often than on *C. cardiochila* (*n* = 51) (GLMM, *z* = −4.522, *P* < 0.001), with only one pollinator removing a pollinium while on the labellum. Twelve males responding to *C. lowanensis* arrived with pollinia attached, but only seven landed on a flower. Of these landing wasps, two landed on the labellum, one of which deposited pollen.

### What is the site of odour release?

**Fig. 4.**
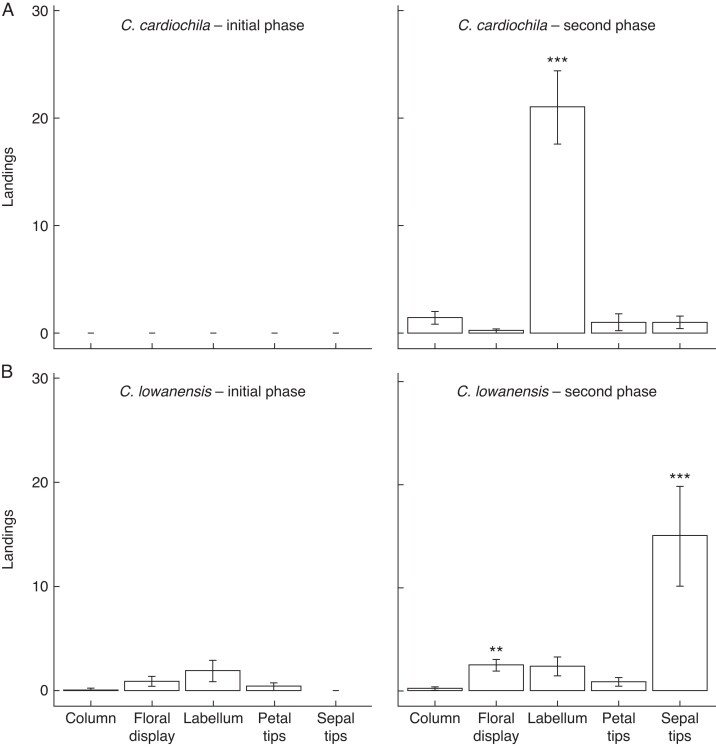
Number of pollinators landing on dissected floral parts of (A) *C. cardiochila* and (B) *C. lowanensis* during the initial phase (only the least attractive floral parts are presented) and the second phase (all floral parts are presented) of the experiment. Means and standard errors for wasp landings per flower are displayed. **Significant difference at *P* < 0.01 between species during the second presentation when all floral parts were presented together; ***P < 0.001.

In our floral manipulation experiments that employed dissections to determine the source of the sexual attractant, clear differences between *C. cardiochila* and *C. lowanensis* were observed that aligned with pollinator behaviour on intact flowers. For *C. cardiochila* (*n* = 5 flowers, 123 wasp responses; Fig. 4A), all males landed during the second experimental phase when the attractive labellum was presented along with the other floral parts. Most wasps responding to *C. cardiochila* landed on the labellum (*n* = 105, mean ± s.e. 21 ± 3.4 per flower), with very few landing on the other floral parts, including the column (*n* = 7, mean ± s.e. 1.4 ± 0.6), petal tips (*n* = 5, mean ± s.e. 1 ± 0.8), sepal tips (*n* = 5, mean ± s.e. 1 ± 0.5) and the floral display (*n* = 1, *mean* ± s.e. 0.2 ± 0.2). One male wasp attempted to mate with the dissected labellum of *C. cardiochila* (1 % of all wasps landing on the labellum). An additional dissection experiment to determine if pollinators are responding predominantly to the thickened labellum tip or to the rest of the labellum that bears insectiform calli (*n* = 6 flowers, 31 wasp responses) revealed no difference in the number of pollinators landing on the dissected labellum tip (*n* = 19, mean ± s.e. 3.2 ± 1) compared with the rest of the labellum (*n* = 12, mean ± s.e. 2 ± 0.6) (GLMM, *z* = 0.378, *P* = 0.705).

For *C. lowanensis* (*n* = 8 flowers, 194 wasp responses; Fig. 4B), 26 wasps landed during the initial phase of the experiment, including on the labellum (*n* = 14 wasps, mean ± s.e. 1.8 ± 1 per flower), floral display (*n* = 8, mean ± s.e. 1 ± 0.5), petal tips (*n* = 3, mean ± s.e. 0.4 ± 0.4) and the column (*n* = 1, mean ± s.e. 0.1 ± 0.1). The remaining 168 wasps all landed during the second phase, which included the attractive sepal tips along with the other floral parts. In this second phase, wasps predominantly landed on the sepal tips (*n* = 120, mean ± s.e. 15 ± 5.3), with some wasps landing on other floral parts including the floral display (*n* = 20, mean ± s.e. 2.5 ± 0.9), labellum (*n* = 19, mean ± s.e. 2.4 ± 0.8), petal tips (*n* = 7, mean ± s.e. 0.9 ± 0.3) and the column (*n* = 2, mean ± s.e. 0.3 ± 0.1). Ten wasps attempted to mate with the dissected sepal clubs (8 % of all wasps that landed on the sepal clubs), while one male displayed non-mating behaviour on the calli of a dissected labellum. During the second phase of the experiment, when all floral parts were presented and wasps’ responses were greatest, significantly more wasps landed on the labella of *C. cardiochila* than the labella of *C. lowanensis* (GLMM, *z* = 10.838, *P* < 0.001; Fig. 4A). In contrast, more wasps landed on the sepal tips (GLMM, *z* = 8.047, *P* < 0.001; Fig. 4B) and the floral display (GLMM, *z* = 2.749, *P* = 0.006; Fig. 4B) of *C. lowanensis* than the sepal tips and floral display of *C. cardiochila*. There were no significant differences in pollinators landing on the column and the petal tips of *C. lowanensis* and *C. cardiochila*.

### Do *C. cardiochila* and *C. lowanensis* provide any sugar?

There were no nectar droplets visible on the labella of either species during sampling. We did not detect the presence of sugar in any of the *C. cardiochila* flowers with GC–MS analysis (*n* = 10). For *C. lowanensis*, however, the disaccharide sucrose (mean ± s.e. 3.66 ± 2.39, range = 0–10.62 µg surface sucrose per flower) as well as monosaccharides (mean ± s.e. 6.18 ± 1.93, range = 0–14.13 µg surface monosaccharides per flower) were detected on the labellum (*n* = 10) at a ratio of 0.6 sucrose to monosaccharides.

### What is the pollination success of *C. cardiochila* and *C. lowanensis* in the field?

Pollination success in *C. cardiochila* varied between the three years, with both the highest (61 % pollinia removal and 29 % pollen deposition) and the lowest (22 % pollinia removal and 11 % pollen deposition) pollination success observed at the MGC site in 2024 and 2023, respectively (Table 2). The pollination efficiency was broadly comparable across years and sites for *C. cardiochila*, ranging from 0.37 to 0.5. Climatic conditions were substantially drier in 2023 (annual rainfall = 476.0 mm) and 2024 (296.8 mm) than in 2022 (568.6 mm), with the number of *C. cardiochila* flowers available for pollination approximately only a third of the numbers observed in 2022. This pattern was especially pronounced in *C. lowanensis*, which only flowered at the KFF site during 2022. While *C. lowanensis* had pollination data for only one year compared to three years for *C. cardiochila*, it is interesting to note that the rate of pollinia removal in *C. lowanensis* (28 %) was lower than that of *C. cardiochila* (mean = 41 %). The rate of pollen deposition (11 %) in *C. lowanensis* and its pollen transfer efficiency (0.4), however, were broadly comparable to *C. cardiochila.* All plants across sites and years produced a single flower, except for five *C. cardiochila* plants that had two flowers in 2022.

**Table 2.** Rates of natural reproductive success at *C. cardiochila* and *C. lowanensis* field sites (*n* refers to the number of flowers available at each site in each year).

Species	Year	Site	Pollinia removed (%)	Pollen deposited (%)	Pollination efficiency
*C. cardiochila*	2022	MGC (*n* = 101)	53.5	22.8	0.43
	2022	BDB (*n* = 121)	26.5	11.6	0.44
	2023	MGC (*n* = 36)	22.2	11.1	0.50
	2023	BDB (*n* = 41)	31.7	14.7	0.46
	2024	MGC (*N* = 38)	60.5	29.0	0.48
	2024	BDB (*n* = 37)	51.4	18.9	0.37
	Average ± s.e.		41 ± 6.6	18 ± 7.0	0.45 ± 0.0
*C. lowanensis*	2022	KFF (*n* = 36)	27.8	11.1	0.40
	2023	KFF (*n* = 0)	NA*	NA*	NA*
	2024	KFF (*n* = 0)	NA*	NA*	NA*

Pollination efficiency refers to the ratio of the number of flowers with pollinia deposited relative the number of flowers with pollinia removed.

^*^During 2023 and 2024 *C. lowanensis* plants did not flower at the KFF site. NA = Non applicable.

## DISCUSSION

We demonstrate here that the morphologically divergent orchids *C. cardiochila* and *C. lowanensis* are dependent on the same sexually deceived thynnine wasp for pollination. *Phymatothynnus* aff. *nitidus* male wasps readily visited both species and were equally likely to display mating behaviour on their flowers, although on different floral parts. *Caladenia cardiochila* exhibits a classic sexually deceptive pollination strategy with its flowers exploiting only mate-seeking male pollinator behaviour without the provision of any potential rewards. *Caladenia lowanensis*, however, also provides a small amount of sugar on the labellum lamina. This suggests that *C. lowanensis* might employ a pollination strategy based on long-range sexual attraction of pollinators followed by a switch to feeding behaviour to lure wasps into the correct position for pollination, as recently described in the closely related *Caladenia robinsonii*, as well as other *Caladenia* species from Western Australia (Phillips *et al.*, 2024*a*). The ability to exploit the same pollinator through sexual deception, despite exhibiting contrasting floral traits like the size and shape of the tepals and labella and the location of sexual attractant, demonstrates the high variability of flower morphology associated with this pollination strategy.

Observations of the behaviour of the shared pollinator of *C. cardiochila* and *C. lowanensis* on their respective flowers revealed striking differences important for pollination. On *C. cardiochila* flowers, pollinators were more likely to land on the labellum and all mating behaviour was directed at the insectiform labellum, either by probing the labellum tip or the calli with the tip of the abdomen. Such pseudocopulation with the labellum by pollinators is frequently reported in other sexually deceptive orchid genera, including *Ophrys* (Paulus, 2006), *Lepanthes* (Blanco and Barboza, 2005), *Pterostylis* (Phillips *et al.*, 2014), *Cryptostylis* (Weinstein *et al.*, 2016) and *Chiloglottis* (de Jager and Peakall, 2016). On *C. cardiochila,* pollen transfer was only observed during such mating behaviour on the labellum, as reported in the Australian *Chiloglottis trapeziformis*, which is also pollinated by sexual deception of thynnine wasps and bears similar insectiform labella with raised calli (de Jager and Peakall, 2019). Only a small proportion of the males attempting to mate with the insectiform labellum in *C. cardiochila* removed pollinia (11.1 % of 45 mating attempts), closely matching the pattern in *Chiloglottis trapeziformis* (12.8 % of 250 mating attempts) (de Jager and Peakall, 2019).

On *C. lowanensis*, pollinators landed on the sepal clubs approximately two-thirds of the time, with approximately a third of wasps landing on the labellum. Mating behaviour was almost entirely directed at the clubs on the sepal tips, far from the reproductive structures in the centre of the flower. This pattern, which is unusual among sexually deceptive orchids, is also reported in other *Caladenia* with the spider orchid floral form that bear clubs (Phillips *et al.*, 2013, 2024; Phillips and Peakall, 2018; Reiter *et al.*, 2023). Contact with the column in *C. lowanensis* was seldom observed and pollen transfer was rare, and never in association with mating behaviour. Instead, column contact and pollen transfer occurred when wasps were focused on the labellum with prolonged contact between their heads and the labellum lamina, where sugar was detected. This behaviour likely reflects a response to food signals, as found in other sexually deceptive *Caladenia*, where sugar is provided on the labellum in addition to the release of sexual attractants from the sepal clubs (Reiter *et al.*, 2023; Phillips *et al.*, 2024*a*).

It is important to note that the amount of sugar provided by most sugar-providing *Caladenia* is small when compared with other nectar-providing orchids (Phillips *et al.*, 2024*a*). Using our methods, a mean of 50 µg of sucrose has been detected per flower in sugar-providing *Caladenia* (mean ± s.e. 49.8 ± 20.8 µg, *n* = 11 species, range 2.6–219.4 µg) (Reiter *et al.*, 2018, 2019*a*, *b*, 2023; Phillips *et al.*, 2020, 2021, 2024*a*; Supplementary Data Fig. S1). While the degree to which these *Caladenia* species can be considered truly rewarding is debatable, these small amounts of sugar have been observed to position wasps for pollination (Phillips *et al.*, 2020, 2024*a*). Apart from *C. lowanensis*, sugars have also been detected in the sexually deceptive *C. xanthochila* (Reiter *et al.*, 2023), *C. robinsonii* and *C. procera* (Phillips *et al.*, 2024*a*). Sugars in *Caladenia* tend to be strongly dominated by sucrose over monosaccharides (Supplementary Data Fig. S1). This sucrose dominance is common across the angiosperms (Chalcoff *et al.*, 2017) and within orchids especially (Lindqvist *et al.*, 2018; Brzosko and Mirski, 2021) and has been found to be preferred by wasp pollinators in some systems (Petanidou, 2005; Brzosko and Mirski, 2021). Interestingly, *C. lowanensis* provides more monosaccharides than sucrose, which is unusual for orchids (Brzosko and Mirski, 2021).

Our floral dissection experiments with *C. lowanensis* and *C. cardiochila* revealed why the stark difference in pollinator landing position was observed when baiting with intact flowers. Once flowers were dissected and wasps offered a choice between floral parts, male wasps nearly always landed on the labellum of *C. cardiochila*, confirming this floral part is the source of the sexual attractant. Interestingly, males were attracted as frequently to the tip of the labellum, which contains a thickened margin of callus tissue, as to the remainder of the labellum, which contains the aggregation of insectiform calli. In contrast, on dissected *C. lowanensis* flowers pollinators most frequently landed on the sepal clubs, revealing that they are the most important source of sexual attractant, as seen in similar *Caladenia* species bearing sepal clubs (Phillips *et al.*, 2013, 2024*a*; Reiter *et al.*, 2023). It is possible, however, based on a low number of pollinator responses, that the labellum and the rest of the floral display produce low levels of sexual attractant in addition to the sepal clubs, as reported in some other *Caladenia* species (Phillips *et al.*, 2024*a*). Alternatively, the combination of nearby sexual odour and a morphological stimulus such as the black dressmaker’s pin that held each flower part in place may also have been sufficient to induce a low frequency of landings.

The pollination rates and pollen transfer efficiency of *C. cardiochila* and *C. lowanensis* observed in this study were largely comparable, despite pollinators being attracted primarily to the sepal clubs on *C. lowanensis*, far from the reproductive structures, with occasional visits to the labellum. Detailed comparisons of reproductive fitness are unfortunately hampered by dry years in 2023 and 2024 when *C. lowanensis* did not flower at the KFF site. *Caladenia cardiochila* also exhibited poor flowering in 2023 and 2024, with approximately only a third of the plants flowering relative to 2022. The average pollination rate (mean ± s.e. 18 ± 7.0 %) and pollinia removal rate (mean ± s.e. 41 ± 6.6 %) for *C. cardiochila* in our study was slightly lower than that of the sexually deceptive and morphologically similar congener *C. tessellata* (28 and 54 %, respectively) (Phillips *et al.*, 2024*b*).

As both *C. cardiochila* and *C. lowanensis* are pollinated by *Phymatothynnus* aff. *nitidus* wasps and overlap in distribution there is potential for hybridization. Indeed, plants with intermediate floral traits have previously been reported in the field, including at the KFF site (Backhouse, 2018). Our crossing experiments further revealed that interspecific crosses between *C. cardiochila* and *C. lowanensis* produced a high percentage of viable seeds, indicating that interspecific pollen transfer events in the field can produce many viable hybrid seeds. While the survival of these hybrid seeds at later life stages is currently unknown, the substantial viability of hybrid seed suggests wild hybrids may form where *C. lowanensis* co-occurs with *C. cardiochila*. Hybridization and backcrossing appear to be common in some congeneric orchids with overlapping distributions (Arduino *et al.*, 1996; Stökl *et al.*, 2008; Pinheiro *et al.*, 2010). Apart from the potential loss of genetic distinctiveness of a given species (Ellstrand and Elam, 1993; Vilà *et al.*, 2000; Pinheiro *et al.*, 2010), hybridization with congeners can also be costly in terms of wasted gametes, especially for rare species (Burgess *et al.*, 2008). When hybrids are sterile or of low fitness, their presence can further reduce the population growth of parental species, which may undergo population decline through demographic swamping (Levin *et al.*, 1996; Prentis *et al.*, 2007). Hybridization in sexually deceptive *Ophrys* orchids, for instance, can lead to severe introgression and a reduction in the number of pure individuals, with the eventual displacement of the rarer parental species likely (Stökl *et al.*, 2008). Some *Ophrys* species that share pollinators reduce their risk of hybridization through divergence in pollinia and stigma morphology (Gögler *et al.*, 2015). While this scenario has not been explicitly investigated in *Caladenia*, it is unlikely to offer protection from hybridization as pollinia in the subgenus *Calonema* are morphologically similar among species (except for some variation in size), and all species studied thus far place their pollinia on the dorsal side of the pollinator’s thorax (Phillips *et al.*, 2009).

The consequence of hybridization for the conservation of endangered *Caladenia* species is currently unclear, as it is unknown how often hybrid phenotypes persist in the wild. Many *Caladenia* hybrids that have been reported appear to be *F*_1_ individuals (Stoutamire, 1983), but no genetic studies have been conducted to determine the extent of hybridization within *Caladenia*. Future studies will benefit from investigating the frequency of hybridization in the endangered *C. lowanensis*. Current conservation actions for *C. lowanensis* may entail identifying potential translocation sites where the pollinator of *C. lowanensis* is abundant within the required habitat, but that are distant enough from wild *C. cardiochila* plants to make pollen transfer between the two species unlikely. It would also be of theoretical interest to elucidate the chemical basis of pollinator attraction within this system, as it is currently unclear whether *C. lowanensis* and *C. cardiochila* employ the same semiochemicals to sexually attract their male wasp pollinator, as documented in the sexually deceptive *Chiloglottis* species *C. trapeziformis* and *C. valida* (Schiestl and Peakall, 2005).

## Supplementary Material

mcaf234_Supplementary_Data

## SUPPLEMENTARY DATA

Supplementary data are available at *Annals of Botany* online and consist of the following. Table S1: site code and year of fieldwork, with details of the type of experiment conducted, the *Caladenia* species tested, the number of experimental trials conducted, and whether it is a wild site for either species. Exact locations are withheld as part of working on threatened flora. Experimental observations and flower dissections comprise repeated visits to a site for detailed experimental data collection, while baiting for pollinators entails surveying for the presence of pollinators at a given location. Table S2: site code and date of wasp pollinator collection for barcoding of the mitochondrial *COI* region, including the *Caladenia* species they were captured on, their ID code and their sex. Figure S1: amount of surface sugar of monosaccharides and sucrose detected on the labellum of 17 *Caladenia* species. Methods follow that of Reiter *et al.* (2018). Species that are sexually deceptive are shown in bold. Error bars indicate standard error for the amount of sucrose present. Video S1: male *Phymatothynnus* aff. *nitidus* wasp pollinators displaying mating behaviour on the labellum of *C. cardiochila.*  Video S2: male *Phymatothynnus* aff. *nitidus* wasp pollinators displaying mating behaviour on the sepal clubs of *C. lowanensis.*  Video S3: male *Phymatothynnus* aff. *nitidus* wasp pollinator removing pollinia from a *C. cardiochila* flower.

## AI ASSISTANCE

None was used.

## References

[mcaf234-B1] Ackerman JD, Phillips RD, Tremblay RL, et al 2023. Beyond the various contrivances by which orchids are pollinated: global patterns in orchid pollination biology. Botanical Journal of the Linnean Society 202: 295–324. doi:10.1093/botlinnean/boac082

[mcaf234-B2] Alcock J . 1981. Notes on the reproductive behavior of some Australian thynnine wasps (Hymenoptera: Tiphiidae). Journal of the Kansas Entomological Society 54: 681–693. http://www.jstor.org/stable/25084214

[mcaf234-B3] Arduino P, Verra F, Cianchi R, Rossi W, Corrias B, Bullini L. 1996. Genetic variation and natural hybridization between *Orchis laxiflora* and *Orchis palustris* (Orchidaceae). Plant Systematics and Evolution 202: 87–109. doi:10.1007/BF00985819

[mcaf234-B4] Australian Government. 2025 . EPBC Act list of threatened flora. https://environment.gov.au/cgi-bin/sprat/public/publicthreatenedlist.pl?wanted=flora (date last accessed January 2025).

[mcaf234-B5] Backhouse G . 2018. Spider orchids: the genus Caladenia and its relatives in Australia. Melbourne: Gary Backhouse.

[mcaf234-B6] Bates R . 1996. Report on the results of field investigations of pollinators of the genus Caladenia (Orchidaceae: Caladeniinae) in 1994–1995. Melbourne: Australian Orchid Foundation.

[mcaf234-B7] Bates D, Mächler M, Bolker B, Walker S. 2015. Fitting linear mixed-effects models using lme4. Journal of Statistical Software 67: 1–48. doi:10.18637/jss.v067.i01

[mcaf234-B8] Blanco M, Barboza G. 2005. Pseudocopulatory pollination in *Lepanthes* (Orchidaceae: Pleurothallidinae) by fungus gnats. Annals of Botany 95: 763–772. doi:10.1093/aob/mci090 15728665 PMC4246739

[mcaf234-B9] Bohman B, Flematti GR, Barrow RA, Pichersky E, Peakall R. 2016. Pollination by sexual deception – it takes chemistry to work. Current Opinion in Plant Biology 32: 37–46. doi:10.1016/j.pbi.2016.06.004 27368084

[mcaf234-B10] Bohman B, Phillips RD, Menz MH, et al 2014. Discovery of pyrazines as pollinator sex pheromones and orchid semiochemicals: implications for the evolution of sexual deception. New Phytologist 203: 939–952. doi:10.1111/nph.12800 24697806

[mcaf234-B11] Brown GR, Phillips RD. 2014. A review of the diet of flower wasps (Hymenoptera: Thynnidae: Thynninae). Northern Territory Naturalist 25: 50–63. doi:10.5962/p.295450

[mcaf234-B12] Brzosko E, Mirski P. 2021. Floral nectar chemistry in orchids: a short review and meta-analysis. Plants 10: 2315. doi:10.3390/plants10112315 34834677 PMC8620889

[mcaf234-B13] Burgess KS, Morgan M, Husband BC. 2008. Interspecific seed discounting and the fertility cost of hybridization in an endangered species. New Phytologist 177: 276–284. doi:10.1111/j.1469-8137.2007.02244.x 17944826

[mcaf234-B14] Carr G. 1991. New taxa in Caladenia R.Br., Chiloglottis R.Br. and Gastrodia R.Br. (Orchidaceae) from south eastern Australia. *Australia Indigenous Flora and Fauna Association Miscellaneous Paper,* No. 1. p. 9.

[mcaf234-B15] Chalcoff VR, Gleiser G, Ezcurra C, Aizen MA. 2017. Pollinator type and secondarily climate are related to nectar sugar composition across the angiosperms. Evolutionary Ecology 31: 585–602. doi:10.1007/s10682-017-9887-2

[mcaf234-B16] Cozzolino S, Nardella A, Impagliazzo S, Widmer A, Lexer C. 2006. Hybridization and conservation of Mediterranean orchids: should we protect the orchid hybrids or the orchid hybrid zones? Biological Conservation 129: 14–23. doi:10.1016/j.biocon.2005.09.043

[mcaf234-B17] de Jager ML, Ellis AG. 2013. The influence of pollinator phylogeography and mate preference on floral divergence in a sexually deceptive daisy. Evolution 67: 1706–1714. doi:10.1111/evo.12070 23730763

[mcaf234-B18] de Jager ML, Ellis AG. 2014. Costs of deception and learned resistance in deceptive interactions. Proceedings of the Royal Society B 281: 20132861. doi:10.1098/rspb.2013.2861 24478302 PMC3924078

[mcaf234-B19] de Jager ML, Newman E, Theron G, Botha P, Barton M, Anderson B. 2016. Pollinators can prefer rewarding models to mimics: consequences for the assumptions of Batesian floral mimicry. Plant Systematics and Evolution 302: 409–418. doi:10.1007/s00606-015-1276-0

[mcaf234-B20] de Jager ML, Peakall R. 2016. Does morphology matter? An explicit assessment of floral morphology in sexual deception. Functional Ecology 30: 537–546. doi:10.1111/1365-2435.12517

[mcaf234-B21] de Jager ML, Peakall R. 2019. Experimental examination of pollinator-mediated selection in a sexually deceptive orchid. Annals of Botany 123: 347–354. doi:10.1093/aob/mcy083 29878057 PMC6344214

[mcaf234-B22] Dixon KW, Hopper SD. 2009. An introduction to *Caladenia* R.Br. – Australasia’s jewel among terrestrial orchids. Australian Journal of Botany 57: ii–viii. doi:10.1071/BT09999

[mcaf234-B23] Ellis AG, Brockington SF, de Jager ML, Mellers G, Walker RH, Glover BJ. 2014. Floral trait variation and integration as a function of sexual deception in *Gorteria diffusa*. Philosophical Transactions of the Royal Society B 369: 20130563. doi:10.1098/rstb.2013.0563 PMC4084545 25002705

[mcaf234-B24] Ellis AG, Johnson SD. 2010. Floral mimicry enhances pollen export: the evolution of pollination by sexual deceit outside of the Orchidaceae. American Naturalist 176: E143–E151. doi:10.1086/656487 20843263

[mcaf234-B25] Ellstrand NC, Elam RD. 1993. Population genetic consequences of small population size: implications for plant conservation. Annual Review of Ecology and Systematics 24: 217–242. doi:10.1146/annurev.es.24.110193.001245

[mcaf234-B26] Fenster CB, Armbruster WS, Wilson P, Dudash MR, Thomson JD. 2004. Pollination syndromes and floral specialization. Annual Review of Ecology, Evolution, and Systematics 35: 375–403. doi:10.1146/annurev.ecolsys.34.011802.132347

[mcaf234-B27] Folmer O, Black M, Hoeh W, Lutz R, Vrijenhoek R. 1994. DNA primers for amplification of mitochondrial cytochrome c oxidase subunit I from diverse metazoan invertebrates. Molecular Marine Biology and Biotechnology 3: 294–299. 7881515

[mcaf234-B28] Gaskett AC . 2012. Floral shape mimicry and variation in sexually deceptive orchids with a shared pollinator. Biological Journal of the Linnean Society 106: 469–481. doi:10.1111/j.1095-8312.2012.01902.x

[mcaf234-B29] Gögler J, Stökl J, Cortis P, et al 2015. Increased divergence in floral morphology strongly reduces gene flow in sympatric sexually deceptive orchids with the same pollinator. Evolutionary Ecology 29: 703–717. doi:10.1007/s10682-015-9779-2

[mcaf234-B30] Griffiths JWH, Trueman G, Brown R, Peakall R. 2011. Molecular genetic analysis and ecological evidence reveals multiple cryptic species among thynnine wasp pollinators of sexually deceptive orchids. Molecular Phylogenetics and Evolution 59: 195–205. doi:10.1016/j.ympev.2011.02.004 21310250

[mcaf234-B31] Harder LD, Johnson SD. 2009. Darwin’s beautiful contrivances: evolutionary and functional evidence for floral adaptation. New Phytologist 183: 530–545. doi:10.1111/j.1469-8137.2009.02914.x 19552694

[mcaf234-B32] Hartig F . 2022. DHARMa: Residual Diagnostics for Hierarchical (Multi-Level/Mixed) Regression Models.

[mcaf234-B33] Hayashi T, Bohman B, Scaffidi A, Peakall R, Flematti G. 2021. An unusual tricosatriene is crucial or male fungus gnat attraction and exploitation by sexually deceptive *Pterostylis* orchids. Current Biology 31: 1954–1961.e7. doi:10.1016/j.cub.2021.01.095 33770489

[mcaf234-B34] Hetherington-Rauth MC, Ramírez SR. 2016. Evolution and diversity of floral scent chemistry in the euglossine bee-pollinated orchid genus *Gongora*. Annals of Botany 118: 135–148. doi:10.1093/aob/mcw072 27240855 PMC4934395

[mcaf234-B35] Hopper SD . 2018. Natural hybridization in the context of Ocbil theory. South African Journal of Botany 118: 284–289. doi:10.1016/j.sajb.2018.02.410

[mcaf234-B36] Johnson SD, Schiestl FP. 2016. Floral mimicry. Oxford: Oxford University Press.

[mcaf234-B37] Johnson SD, Steiner KE. 1997. Long-tongued fly pollination and evolution of floral spur length in the *Disa draconis* complex (Orchidaceae). Evolution 51: 45–53. doi:10.2307/2410959 28568792

[mcaf234-B38] Jones DL . 2021. Complete guide to native orchids of Australia. Sydney: New Holland.

[mcaf234-B39] Kopper C, Schönenberger J, Dellinger AS. 2024. High floral disparity without pollinator shifts in buzz-bee-pollinated Melastomataceae. New Phytologist 242: 2322–2337. doi:10.1111/nph.19735 38634161

[mcaf234-B40] Levin DA, Francisco-Ortega J, Jansen RK. 1996. Hybridization and the extinction of rare plant species. Conservation Biology 10: 10–16. doi:10.1046/j.1523-1739.1996.10010010.x

[mcaf234-B41] Lindqvist DN, Pedersen H, Rasmussen LH. 2018. A novel technique for determination of the fructose, glucose and sucrose distribution in nectar from orchids by HPLC-ELSD. Journal of Chromatography B 1081–1082: 126–130. doi:10.1016/j.jchromb.2018.02.019 29524853

[mcaf234-B42] Menz MH, Brown GR, Dixon KW, Phillips RD. 2015. Absence of nectar resource partitioning in a community of parasitoid wasps. Journal of Insect Conservation 19: 703–711. doi:10.1007/s10841-015-9793-2

[mcaf234-B43] Menz MH, Phillips RD, Dixon KW, Peakall R, Didham RK. 2013. Mate-searching behaviour of common and rare wasps and the implications for pollen movement of the sexually deceptive orchids they pollinate. PLoS One 8: e59111. doi:10.1371/journal.pone.0059111 23536860 PMC3594162

[mcaf234-B44] Minnaar C, Anderson B, de Jager ML, Karron JD. 2019. Plant–pollinator interactions along the pathway to paternity. Annals of Botany 123: 225–245. doi:10.1093/aob/mcy167 30535041 PMC6344347

[mcaf234-B45] Newman E, Anderson B, Johnson SD. 2012. Flower colour adaptation in a mimetic orchid. Proceedings of the Royal Society B 279: 2309–2313. doi:10.1098/rspb.2011.2375 22298842 PMC3350669

[mcaf234-B46] Ollerton J, Winfree R, Tarrant S. 2011. How many flowering plants are pollinated by animals? Oikos 120: 321–326. doi:10.1111/j.1600-0706.2010.18644.x

[mcaf234-B47] Paul J, Budd C, Freeland JR. 2013. Conservation genetics of an endangered orchid in eastern Canada. Conservation Genetics 14: 195–204. doi:10.1007/s10592-012-0443-x

[mcaf234-B48] Paulus HF . 2006. Deceived males – pollination biology of the Mediterranean orchid genus *Ophrys* (Orchidaceae). Journal Europäischer Orchideen 38: 303–351.

[mcaf234-B49] Pauw A, Stofberg J, Waterman RJ. 2009. Flies and flowers in Darwin’s race. Evolution 63: 268–279. doi:10.1111/j.1558-5646.2008.00547.x 19146595

[mcaf234-B50] Peakall R . 1990. Responses of male *Zaspilothynnus trilobatus* Turner wasps to females and the sexually deceptive orchid it pollinates. Functional Ecology 4: 159–167. doi:10.2307/2389335

[mcaf234-B51] Peakall R . 2023. Pollination by sexual deception. Current Biology 33: R489–R496. doi:10.1016/j.cub.2023.02.066 37279681

[mcaf234-B52] Peakall R, Beattie AJ. 1996. Ecological and genetic consequences of pollination by sexual deception in the orchid *Caladenia tentaculata*. Evolution 50: 2207–2220. doi:10.2307/2410692 28565662

[mcaf234-B53] Peakall R, Ebert D, Poldy J, et al 2010. Pollinator specificity, floral odour chemistry and the phylogeny of Australian sexually deceptive *Chiloglottis* orchids: implications for pollinator-driven speciation. New Phytologist 188: 437–450. doi:10.1111/j.1469-8137.2010.03308.x 20561345

[mcaf234-B54] Petanidou T . 2005. Sugars in Mediterranean floral nectars: an ecological and evolutionary approach. Journal of Chemical Ecology 31: 1065–1088. doi:10.1007/s10886-005-4248-y 16124233

[mcaf234-B55] Phillips RD, Bohman B, Brown GR, Tomlinson S, Peakall R. 2020. A specialised pollination system using nectar-seeking thynnine wasps in *Caladenia nobilis* (Orchidaceae). Plant Biology 22: 157–166. doi:10.1111/plb.13069 31705712

[mcaf234-B56] Phillips RD, Bohman B, Peakall R. 2021. Pollination by nectar-foraging pompilid wasps: a new specialized pollination strategy for the Australian flora. Plant Biology 23: 702–710. doi:10.1111/plb.13286 33998761

[mcaf234-B57] Phillips RD, Bohman B, Peakall R, Reiter N. 2024a. Sexual attraction with pollination during feeding behaviour: implications for transitions between specialized strategies. Annals of Botany 133: 273–286. doi:10.1093/aob/mcad178 37963103 PMC11005785

[mcaf234-B58] Phillips RD, Brown GR, Dixon KW, Hayes C, Linde CC, Peakall R. 2017. Evolutionary relationships among pollinators and repeated pollinator sharing in sexually deceptive orchids. Journal of Evolutionary Biology 30: 1674–1691. doi:10.1111/jeb.13125 28714217

[mcaf234-B59] Phillips RD, Faast R, Bower CC, Brown GR, Peakall R. 2009. Implications of pollination by food and sexual deception for pollinator specificity, fruit set, population genetics and conservation of *Caladenia* (Orchidaceae). Australian Journal of Botany 57: 287–306. doi:10.1071/BT08154

[mcaf234-B60] Phillips RD, Hatley J, Li X, Dimon RJ, Reiter N. 2024b. Resilience to summer bushfire in the threatened orchid, *Caladenia tessellata*, in terms of pollination success, herbivory, and mycorrhizal associations. Botanical Journal of the Linnean Society 205: 365–378. doi:10.1093/botlinnean/boad079

[mcaf234-B61] Phillips RD, Peakall R. 2018. Breaking the rules: discovery of sexual deception in *Caladenia abbreviata* (Orchidaceae), a species with brightly coloured flowers and a non-insectiform labellum. Australian Journal of Botany 66: 95–100. doi:10.1071/BT17151

[mcaf234-B62] Phillips RD, Scaccabarozzi D, Retter BA, et al 2014. Caught in the act: pollination of sexually deceptive trap-flowers by fungus gnats in *Pterostylis* (Orchidaceae). Annals of Botany 113: 629–641. doi:10.1093/aob/mct295 24366109 PMC3936588

[mcaf234-B63] Phillips RD, Xu T, Hutchinson MF, Dixon KW, Peakall R. 2013. Convergent specialization – the sharing of pollinators by sympatric genera of sexually deceptive orchids. Journal of Ecology 101: 826–835. doi:10.1111/1365-2745.12068

[mcaf234-B64] Pinheiro F, De Barros F, Palma-Silva CL, et al 2010. Hybridization and introgression across different ploidy levels in the Neotropical orchids *Epidendrum fulgens* and *E. puniceoluteum* (Orchidaceae). Molecular Ecology 19: 3981–3994. doi:10.1111/j.1365-294X.2010.04780.x 20807337

[mcaf234-B65] Prentis PJ, White EM, Radford IJ, Lowe AJ, Clarke AR. 2007. Can hybridization cause local extinction: a case for demographic swamping of the Australian native *Senecio pinnatifolius* by the invasive *Senecio madagascariensis*? New Phytologist 176: 902–912. doi:10.1111/j.1469-8137.2007.02217.x 17850249

[mcaf234-B66] Pritchard HW . 1985. Determination of orchid seed viability using fluorescein diacetate. Plant Cell and Environment 8: 727–730. doi:10.1111/1365-3040.ep11611849

[mcaf234-B67] R Core Team . 2016. R: a language and environment for statistical computing. Vienna, Austria: R Foundation for Statistical Computing.

[mcaf234-B68] Reiter N, Bohman B, Batley M, Phillips RD. 2019a. Pollination of an endangered *Caladenia* species (Orchidaceae) by nectar-foraging behaviour of a widespread species of colletid bee. Botanical Journal of the Linnean Society 189: 83–98. doi:10.1093/botlinnean/boy074

[mcaf234-B69] Reiter N, Bohman B, Flematti GR, Phillips RD. 2018. Pollination by nectar-foraging thynnine wasps: evidence of a new specialized pollination system for Australian orchids. Botanical Journal of the Linnean Society 188: 327–337. doi:10.1093/botlinnean/boy058

[mcaf234-B70] Reiter N, Bohman B, Freestone M, Brown GR, Phillips RD. 2019b. Pollination by nectar-foraging thynnine wasps in the endangered *Caladenia arenaria* and *Caladenia concolor* (Orchidaceae). Australian Journal of Botany 67: 490–500. doi:10.1071/BT19033

[mcaf234-B71] Reiter N, Whitfield J, Pollard G, et al 2016. Orchid re-introductions: an evaluation of success and ecological considerations using key comparative studies from Australia. Plant Ecology 217: 81–95. doi:10.1007/s11258-015-0561-x

[mcaf234-B72] Reiter N, Wicks M, Pollard G, Brown GR, Menz MH, Bohman B. 2023. Improving conservation and translocation success of an endangered orchid, *Caladenia xanthochila* (Orchidaceae), through understanding pollination. Plant Ecology 224: 715–727. doi:10.1007/s11258-023-01334-0

[mcaf234-B73] Rosas-Guerrero V, Aguilar R, Martén-Rodríguez S, et al 2014. A quantitative review of pollination syndromes: do floral traits predict effective pollinators? Ecology Letters 17: 388–400. doi:10.1111/ele.12224 24393294

[mcaf234-B74] Schiestl FP, Peakall R. 2005. Two orchids attract different pollinators with the same floral odour compound: ecological and evolutionary implications. Functional Ecology 19: 674–680. doi:10.1111/j.1365-2435.2005.01010.x

[mcaf234-B75] Schiestl FP, Peakall R, Mant JG, et al 2003. The chemistry of sexual deception in an orchid-wasp pollination system. Science 302: 437–438. doi:10.1126/science.1087835 14564006

[mcaf234-B76] Sletvold N, Trunschke J, Smit M, Verbeek J, Ågren J. 2016. Strong pollinator-mediated selection for increased flower brightness and contrast in a deceptive orchid. Evolution 70: 716–724. doi:10.1111/evo.12881 26878831

[mcaf234-B77] Stökl J, Schlüter PM, Stuessy T, Paulus HF, Assum G, Ayasse M. 2008. Scent variation and hybridization cause the displacement of a sexually deceptive orchid species. American Journal of Botany 95: 472–481. doi:10.3732/ajb.95.4.472 21632372

[mcaf234-B78] Stoutamire W . 1974. Australian terrestrial orchids, thynnid wasps and pseudocopulation. American Orchid Society Bulletin 43: 13–18.

[mcaf234-B79] Stoutamire W . 1983. Wasp-pollinated species of *Caladenia* (Orchidaceae) in south-western Australia. Australian Journal of Botany 31: 383–394. doi:10.1071/BT9830383

[mcaf234-B80] Swarts ND, Clements MA, Bower CC, Miller JT. 2014. Defining conservation units in a complex of morphologically similar, sexually deceptive, highly endangered orchids. Biological Conservation 174: 55–64. doi:10.1016/j.biocon.2014.03.017

[mcaf234-B81] Tate R . 1887. Transactions and proceedings and report of the Royal Society of South Australia. Adelaide, Australia, The Society, 1880-1889 9: 60–61.

[mcaf234-B82] Todd JA. 2000. *Recovery plan for twelve threatened Spider-orchid Caladenia taxa (Orchidaceae: Caladeniinae) of Victoria and South Australia 2000-2004*. Melbourne: Department of Natural Resources and Environment.

[mcaf234-B83] Tremblay RL, Ackerman JD, Zimmerman JK, Calvo RN. 2005. Variation in sexual reproduction in orchids and its evolutionary consequences: a spasmodic journey to diversification. Biological Journal of the Linnean Society 84: 1–54. doi:10.1111/j.1095-8312.2004.00400.x

[mcaf234-B84] VicFlora . 2025. Flora of Victoria. Melbourne: Royal Botanic Gardens Victoria.

[mcaf234-B85] Vilà M, Weber E, Antonio CMD. 2000. Conservation implications of invasion by plant hybridization. Biological Invasions 2: 207–217. doi:10.1023/A:1010003603310

[mcaf234-B86] Weinstein AM, Davis BJ, Menz MH, Dixon KW, Phillips RD. 2016. Behaviour of sexually deceived ichneumonid wasps and its implications for pollination in *Cryptostylis* (Orchidaceae). Biological Journal of the Linnean Society 119: 283–298. doi:10.1111/bij.12841

[mcaf234-B87] Whitehead MR, Peakall R. 2013. Short-term but not long-term patch avoidance in an orchid-pollinating solitary wasp. Behavioral Ecology 24: 162–168. doi:10.1093/beheco/ars149

[mcaf234-B88] Whitehead MR, Peakall R. 2014. Pollinator specificity drives strong prepollination reproductive isolation in sympatric sexually deceptive orchids. Evolution 68: 1561–1575. doi:10.1111/evo.12382 24527666

[mcaf234-B89] Widholm JM . 1972. The use of fluorescein diacetate and phenosafranine for determining the viability of cultured plant cells. Stain Technology 47: 189–194. doi:10.3109/10520297209116483 4113995

[mcaf234-B90] Wong DC, Amarasinghe R, Rodriguez-Delgado C, Eyles R, Pichersky E, Peakall R. 2017. Tissue-specific floral transcriptome analysis of the sexually deceptive orchid *Chiloglottis trapeziformis* provides insights into the biosynthesis and regulation of its unique UV-B dependent floral volatile, chiloglottone 1. Frontiers in Plant Science 8: 1260. doi:10.3389/fpls.2017.01260 28769963 PMC5515871

[mcaf234-B91] Xu S, Schlüter PM, Scopece G, et al 2011. Floral isolation is the main reproductive barrier among closely related sexually deceptive orchids. Evolution 65: 2606–2620. doi:10.1111/j.1558-5646.2011.01323.x 21884059

