# Supplementary material for: Sexually deceptive orchids with distinct flower morphologies elicit different behaviours from a shared pollinator
Source: Ann Bot. 2025 Sep 26;137(1):281–94. doi: 10.1093/aob/mcaf234 (PMC12784070; doi:10.1093/aob/mcaf234)
Supplement: mcaf234_Supplementary_Data [file mcaf234_supplementary_data.zip › Supplementary Data.pdf]

Supplementary Table 1. Site code and year of fieldwork, with details of the type of experiment conducted, the *Caladenia* species tested, the number of experimental trials conducted, and whether it is a wild site for either species. Exact locations withheld as part of working on threatened flora. Experimental observations and flower dissections comprise repeated visits to a site for detailed experimental data collection, while baiting for pollinators entail surveying for the presence of pollinators at a given location.

| Site code | Year | Experiment | Species tested | Trials | Wild site for |
|---|---|---|---|---|---|
| MGC | 2022 | Experimental observations | *C. lowanensis* | 90 x 3min | *C. cardiochila* |
| MGC | 2022 | Experimental observations | *C. cardiochila* | 90 x 3min | *C. cardiochila* |
| MGC | 2022 | Flower dissections | *C. lowanensis* | 30 x 3min | *C. cardiochila* |
| MGC | 2022 | Flower dissections | *C. cardiochila* | 40 x 3min | *C. cardiochila* |
| MGC | 2023 | Experimental observations | *C. lowanensis* | 25 x 3min | *C. cardiochila* |
| MGC | 2023 | Experimental observations | *C. cardiochila* | 25 x 3min | *C. cardiochila* |
| MGC | 2023 | Flower dissections | *C. lowanensis* | 10 x 3min | *C. cardiochila* |
| MGC | 2023 | Flower dissections | *C. cardiochila* | 6 x 3min | *C. cardiochila* |
| MGC | 2024 | Experimental observations | *C. lowanensis* | 5 x 3min | *C. cardiochila* |
| MGC | 2024 | Experimental observations | *C. cardiochila* | 5 x 3min | *C. cardiochila* |
| RG | 2023 | Baiting for pollinators | *C. lowanensis* | 4 x10min | |
| RG | 2023 | Baiting for pollinators | *C. cardiochila* | 4 x10min | |
| BDB | 2022 | Baiting for pollinators | *C. lowanensis* | 4 x10min | *C. cardiochila* |
| BDB | 2022 | Baiting for pollinators | *C. cardiochila* | 4 x10min | *C. cardiochila* |
| BDB | 2024 | Baiting for pollinators | *C. lowanensis* | 4 x10min | *C. cardiochila* |
| BDB | 2024 | Baiting for pollinators | *C. cardiochila* | 4 x10min | *C. cardiochila* |
| KFF | 2022 | Baiting for pollinators | *C. lowanensis* | 6 x10min | *C. lowanensis* |
| KFF | 2022 | Baiting for pollinators | *C. cardiochila* | 6 x10min | *C. lowanensis* |
| KFF | 2023 | Baiting for pollinators | *C. lowanensis* | 9 x10min | *C. lowanensis* |
| KFF | 2024 | Baiting for pollinators | *C. lowanensis* | 46 x10min | *C. lowanensis* |
| WWFF | 2023 | Baiting for pollinators | *C. lowanensis* | 4 x10min | *C. lowanensis* |
| WWFF | 2024 | Baiting for pollinators | *C. lowanensis* | 27 x10min | *C. lowanensis* |
| BTFN | 2023 | Baiting for pollinators | *C. lowanensis* | 4 x10min | |
| BTFN | 2023 | Baiting for pollinators | *C. cardiochila* | 4 x10min | |
| MATSP | 2023 | Baiting for pollinators | *C. lowanensis* | 6 x10min | |
| MATSP | 2024 | Baiting for pollinators | *C. lowanensis* | 4 x10min | |
| MATSP | 2024 | Baiting for pollinators | *C. cardiochila* | 4 x10min | |
| BNCR | 2023 | Baiting for pollinators | *C. lowanensis* | 2 x10min | |
| WSF | 2023 | Baiting for pollinators | *C. lowanensis* | 1 x10min | |
| GNCR | 2023 | Baiting for pollinators | *C. lowanensis* | 2 x10min | |
| ASF | 2023 | Baiting for pollinators | *C. lowanensis* | 3 x10min | |
| CSR | 2023 | Baiting for pollinators | *C. lowanensis* | 3 x10min | |
| DNCR | 2023 | Baiting for pollinators | *C. lowanensis* | 3 x10min | |

| | | | | |
|---|---|---|---|---|
| DNCR | 2023 | Baiting for pollinators | *C. cardiochila* | 3 x10min |
| DNCR | 2023 | Baiting for pollinators | *C. lowanensis* | 2 x10min |
| LDNP3 | 2023 | Baiting for pollinators | *C. lowanensis* | 4 x10min |
| LDNP3 | 2023 | Baiting for pollinators | *C. cardiochila* | 4 x10min |
| LDNP6 | 2023 | Baiting for pollinators | *C. lowanensis* | 4 x10min |
| LDNP6 | 2023 | Baiting for pollinators | *C. cardiochila* | 4 x10min |
| SMNCR | 2023 | Baiting for pollinators | *C. lowanensis* | 1 x10min |
| HHNCR | 2023 | Baiting for pollinators | *C. lowanensis* | 1 x10min |
| BRSP | 2023 | Baiting for pollinators | *C. lowanensis* | 4 x10min |
| BRSP | 2023 | Baiting for pollinators | *C. cardiochila* | 4 x10min |
| BRSP | 2024 | Baiting for pollinators | *C. lowanensis* | 6 x10min |
| MBR | 2024 | Baiting for pollinators | *C. lowanensis* | 1 x10min |
| LNCR | 2024 | Baiting for pollinators | *C. lowanensis* | 1 x10min |
| DSF | 2023 | Baiting for pollinators | *C. lowanensis* | 2 x10min |
| DSF | 2023 | Baiting for pollinators | *C. cardiochila* | 2 x10min |
| DSF | 2024 | Baiting for pollinators | *C. lowanensis* | 2 x10min |
| DLNCR | 2023 | Baiting for pollinators | *C. lowanensis* | 6 x10min |
| DLNCR | 2023 | Baiting for pollinators | *C. cardiochila* | 6 x10min |
| DLNCR | 2024 | Baiting for pollinators | *C. lowanensis* | 2 x10min |
| SRG | 2024 | Baiting for pollinators | *C. lowanensis* | 6 x10min |
| DUNCR | 2024 | Baiting for pollinators | *C. lowanensis* | 2 x10min |

Supplementary Table 2. Site code and date of wasp pollinators collected for barcoding of the mitochondrial *COI* region, including the *Caladenia* species they were captured on, their ID code and their sex.

| **Site code** | **Date** | **Orchid** | **Pollinator code** | **Sex** |
|---|---|---|---|---|
| MGC | 22.09.2022 | *C. cardiochila* | MGCCC12022 | Male |
| MGC | 22.09.2022 | *C. cardiochila* | MGCCC22022 | Male |
| MGC | 25.09.2022 | *C. cardiochila* | MCGCC32022 | Male |
| MGC | 25.09.2022 | *C. cardiochila* | MGCCC52022 | Male |
| MGC | 14.09.2023 | *C. cardiochila* | MGCCC62023 | Male |
| MGC | 14.09.2023 | *C. cardiochila* | MGCCC82023 | Male |
| MGC | 14.09.2023 | *C. cardiochila* | MGCCCf2023 | Female |
| MGC | 14.09.2023 | *C. cardiochila* | MGCCC92023 | Male |
| MGC | 22.09.2022 | *C. lowanensis* | MGCCL12022 | Male |
| MGC | 25.09.2022 | *C. lowanensis* | MCGCL52022 | Male |
| MGC | 25.09.2022 | *C. lowanensis* | MGCCL72022 | Male |
| MGC | 18.10.2022 | *C. lowanensis* | MGCCL82022 | Male |
| MGC | 18.10.2022 | *C. lowanensis* | MGCCL92022 | Male |
| MGC | 14.09.2023 | *C. lowanensis* | MGCCL102023 | Male |
| MGC | 14.09.2023 | *C. lowanensis* | MGCCL112023 | Male |
| MGC | 14.09.2023 | *C. lowanensis* | MGCCL122023 | Male |
| MGC | 14.09.2023 | *C. lowanensis* | MGCCL132023 | Male |
| MGC | 24.09.2024 | *C. lowanensis* | MGCCL142024 | Male |
| MGC | 24.09.2024 | *C. lowanensis* | MGCCL152024 | Male |
| MGC | 24.09.2024 | *C. lowanensis* | MGCCL162024 | Male |
| MGC | 24.09.2024 | *C. lowanensis* | MGCCL172024 | Male |
| MGC | 24.09.2024 | *C. lowanensis* | MGCCL182024 | Male |
| MGC | 24.09.2024 | *C. cardiochila* | MGCCC142024 | Male |
| MGC | 24.09.2024 | *C. cardiochila* | MGCCC152024 | Male |
| MGC | 24.09.2024 | *C. cardiochila* | MGCCC162024 | Male |
| MGC | 24.09.2024 | *C. cardiochila* | MGCCC172024 | Male |
| MGC | 24.09.2024 | *C. cardiochila* | MGCCC182024 | Male |
| BTFN | 19.09.2023 | *C. lowanensis* | BEL1LOW12023 | Male |
| BTFN | 19.09.2023 | *C. lowanensis* | BEL1LOW22023 | Male |
| BTFN | 19.09.2023 | *C. cardiochila* | BEL1CAR12023 | Male |
| LDNP | 28.09.2023 | *C. lowanensis* | LIT3LOW12023 | Male |
| LDNP | 02.10.2023 | *C. lowanensis* | LIT6LOW22023 | Male |
| MATSP | 29.09.2023 | *C. lowanensis* | MPW32023 | Male |
| MATSP | 29.09.2023 | *C. lowanensis* | MPW42023 | Male |
| MATSP | 29.09.2023 | *C. lowanensis* | MPW52032 | Male |

| | | | | |
|---|---|---|---|---|
| MATSP | 29.09.2023 | *C. lowanensis* | MPW62023 | Male |
| WSF | 30.09.2023 | *C. lowanensis* | MPW122023 | Male |
| ASF | 02.10.2023 | *C. lowanensis* | MPW132023 | Male |
| ASF | 29.09.2023 | *C. lowanensis* | MPW152023 | Male |
| ASF | 29.09.2023 | *C. lowanensis* | MPW172023 | Male |
| KFF | 04.09.2024 | *C. lowanensis* | MPW12024 | Male |

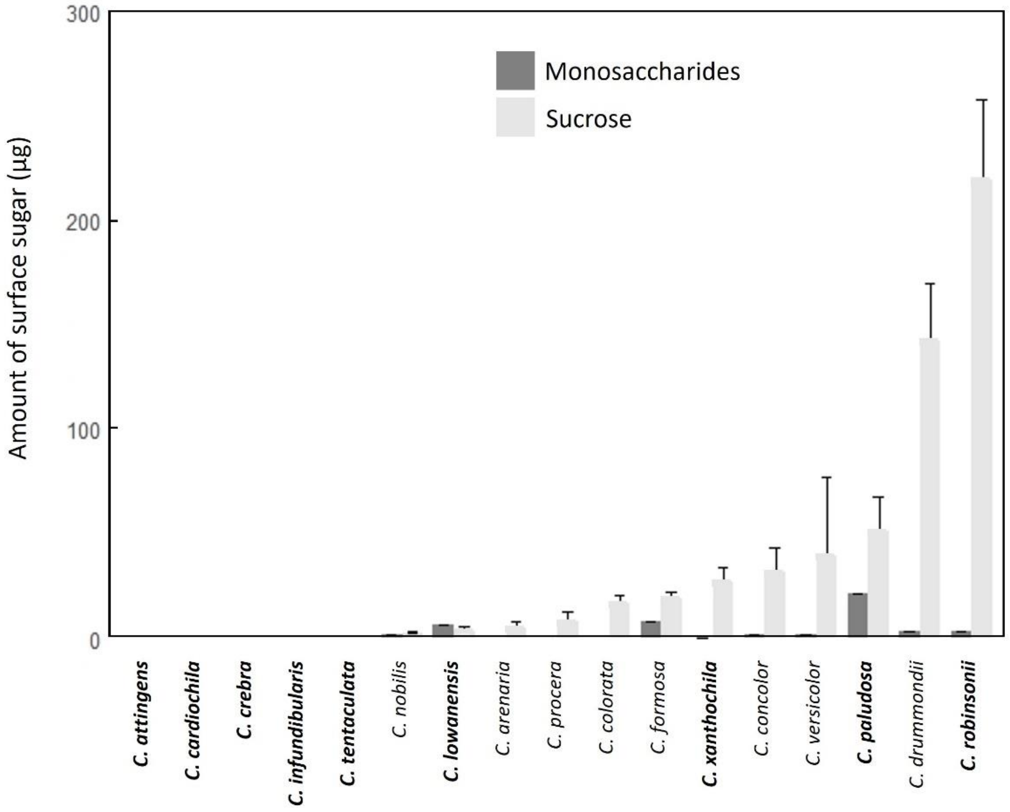


Supplementary Figure 1. The amount of surface sugar of monosaccharides and sucrose detected on the labellum of 17 *Caladenia* species. Methods follow that of Reiter *et al.,* (2018). Species that are sexually deceptive are shown in bold. Error bars indicate standard error for the amount of sucrose present.

*References*


**Reiter N, Bohman B, Flematti GR, Phillips RD. 2018.** Pollination by nectar-foraging thynnine wasps: evidence of a new specialized pollination system for Australian orchids. *Botanical Journal of the Linnean Society* **188:** 327–337.
