# Supplementary figures and images for: Sexually deceptive orchids with distinct flower morphologies elicit different behaviours from a shared pollinator
Source: Ann Bot. 2025 Sep 26;137(1):281–94. doi: 10.1093/aob/mcaf234 (PMC12784070; doi:10.1093/aob/mcaf234)

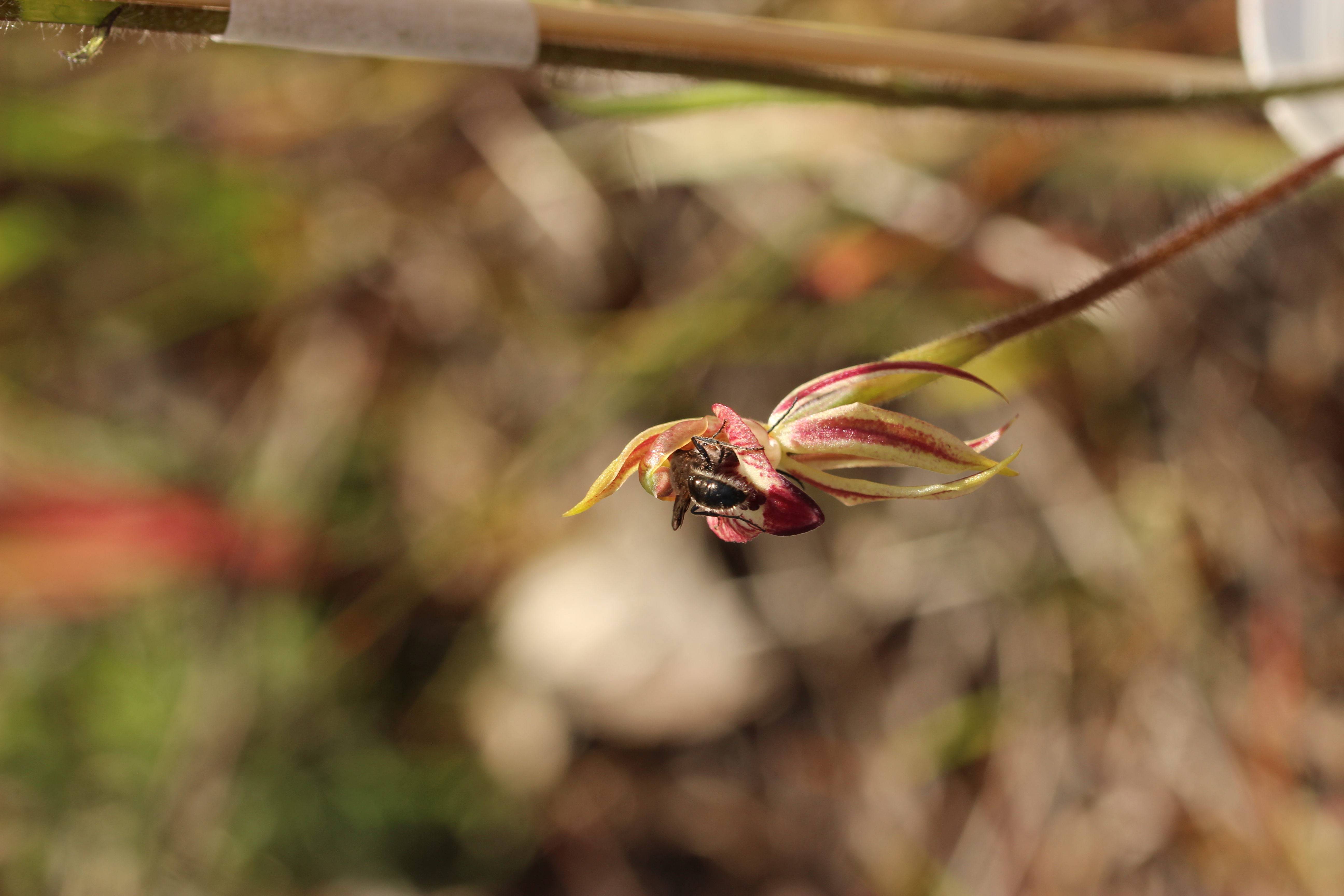

Supplement: mcaf234_Supplementary_Data [file mcaf234_supplementary_data.zip › Fig. 3A.JPG]

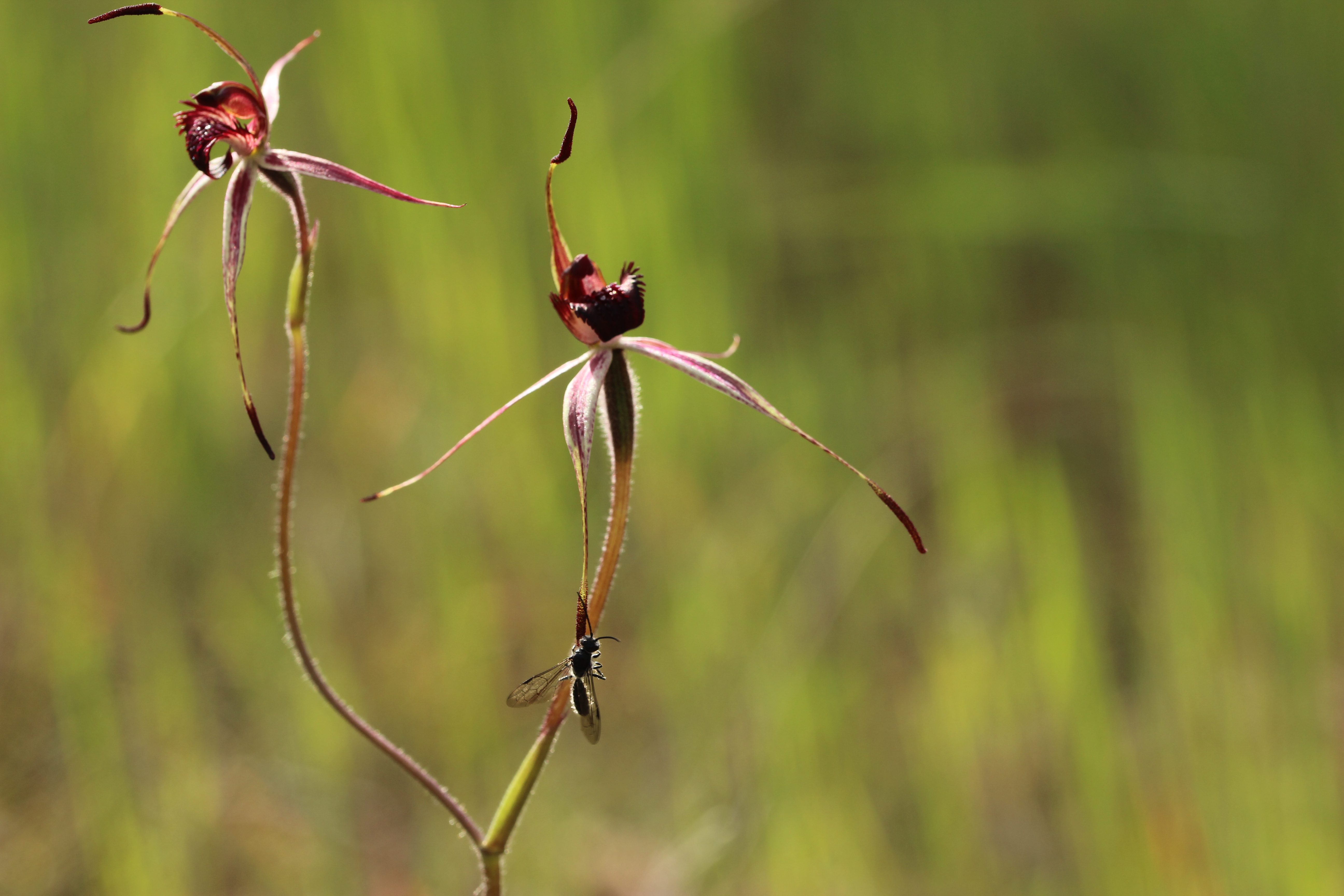

Supplement: mcaf234_Supplementary_Data [file mcaf234_supplementary_data.zip › Fig. 3B.JPG]

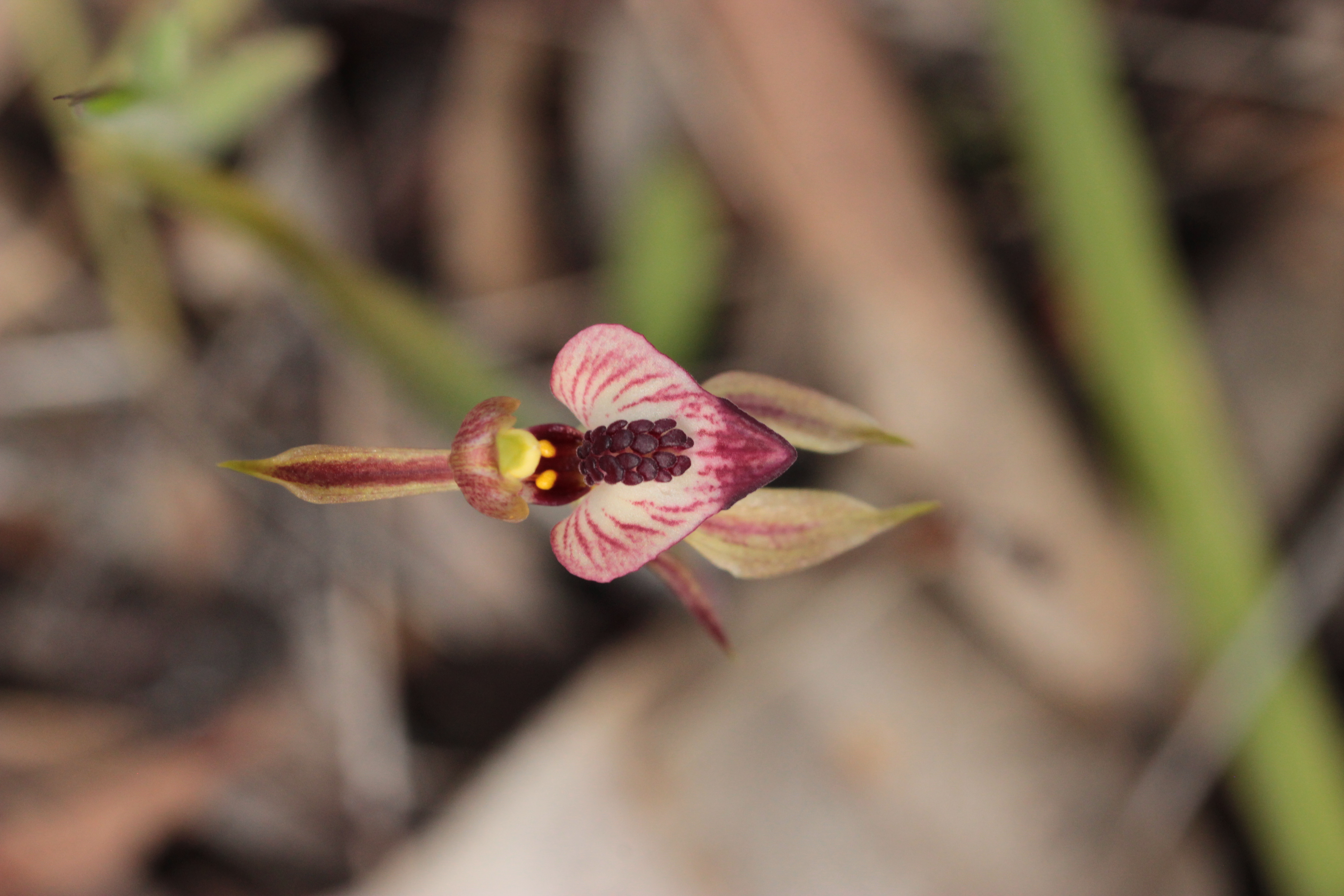

Supplement: mcaf234_Supplementary_Data [file mcaf234_supplementary_data.zip › Fig. 3C.JPG]

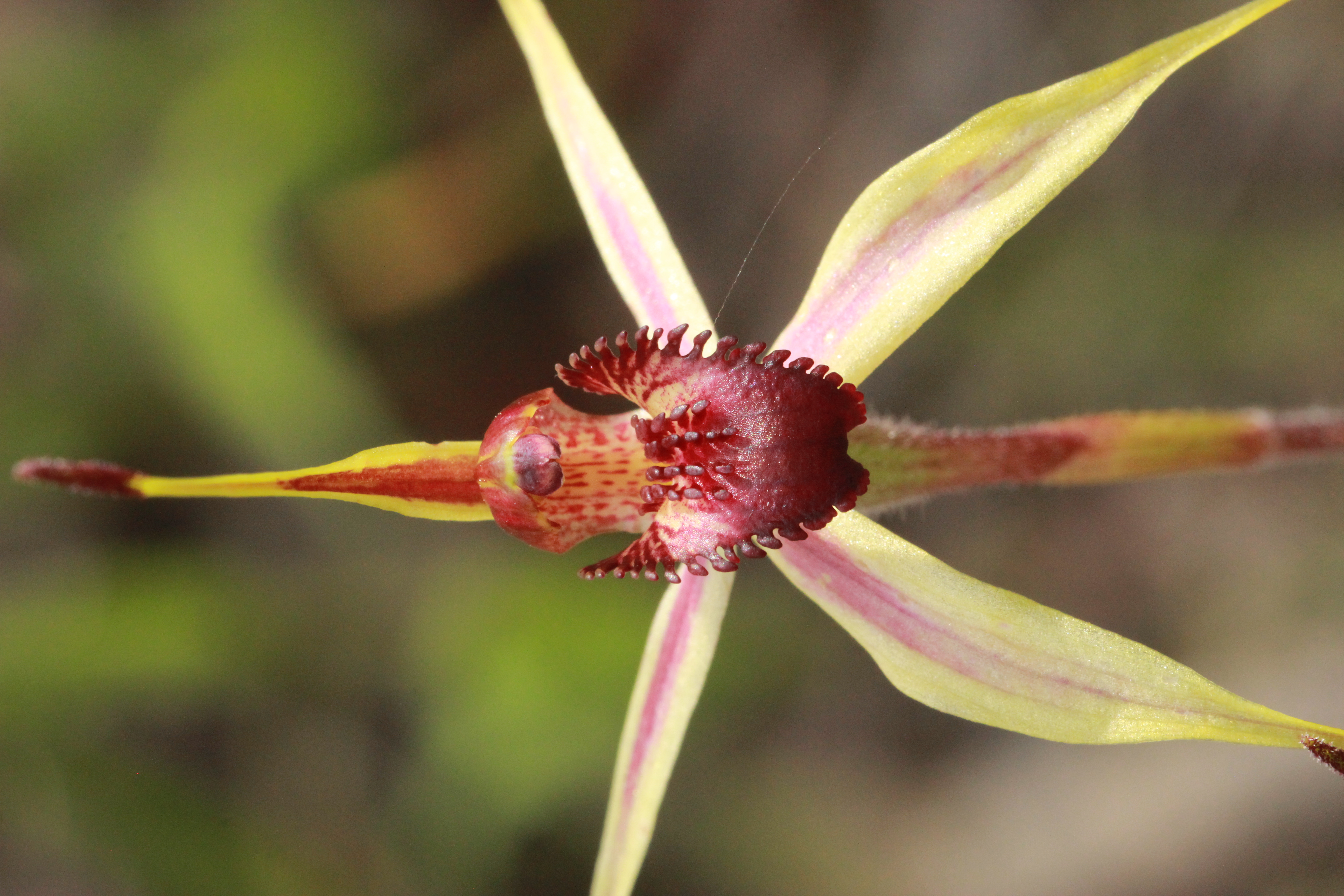

Supplement: mcaf234_Supplementary_Data [file mcaf234_supplementary_data.zip › Fig. 3D.JPG]

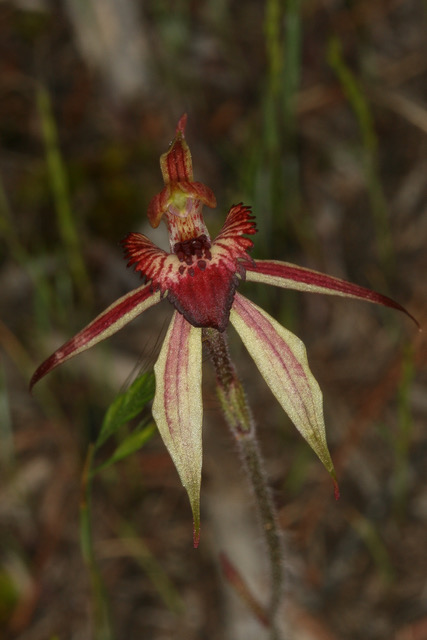

Supplement: mcaf234_Supplementary_Data [file mcaf234_supplementary_data.zip › Fig. 3E.jpeg]
